# Locating Single-Atom Optical Picocavities Using Wavelength-Multiplexed Raman Scattering

**DOI:** 10.1021/acsphotonics.1c01100

**Published:** 2021-10-04

**Authors:** Jack Griffiths, Bart de Nijs, Rohit Chikkaraddy, Jeremy J. Baumberg

**Affiliations:** † NanoPhotonics Centre, Cavendish Laboratory, University of Cambridge, J J Thomson Avenue, Cambridge CB3 0HE, U.K.

**Keywords:** plasmonics, picocavity, adatom, SERS, localization spectroscopy

## Abstract

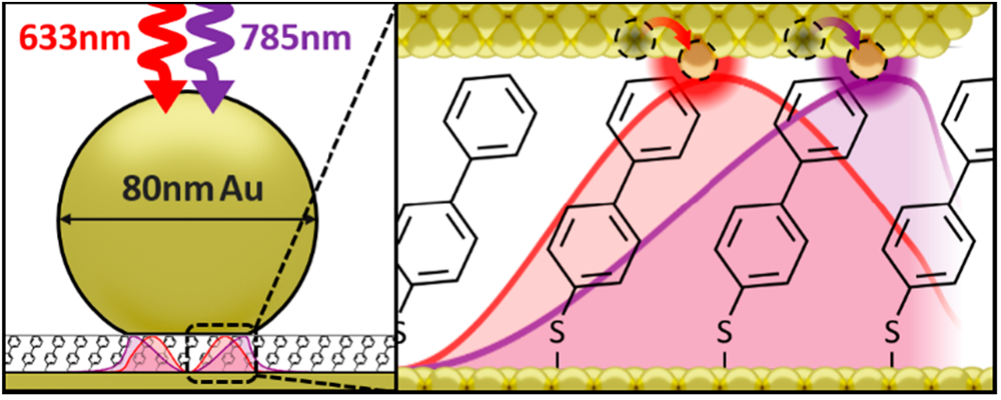

Transient atomic protrusions in plasmonic nanocavities confine optical fields to sub-1-nm^3^ picocavities, allowing the optical interrogation of single molecules at room temperature. While picocavity formation is linked to both the local chemical environment and optical irradiation, the role of light in localizing the picocavity formation is unclear. Here, we combine information from thousands of picocavity events and simultaneously compare the transient Raman scattering arising from two incident pump wavelengths. Full analysis of the data set suggests that light suppresses the local effective barrier height for adatom formation and that the initial barrier height is decreased by reduced atomic coordination numbers near facet edges. Modeling the system also resolves the frequency-dependent picocavity field enhancements supported by these atomic scale features.

Nanostructures of plasmonic metals confine optical fields to subdiffraction-limited gaps between metal surfaces.^1^ The correspondingly magnified field is key for enhancing light–matter interactions such as fluorescence^2^ within these plasmonic nanocavities, even reaching the regime of single molecule light–matter strong coupling at room temperature.^3^ This is especially pertinent for surface enhanced Raman scattering (SERS) which probes the vibrational modes of matter since, despite intrinsically small Raman scattering cross sections, it increases as the fourth power of the field enhancement.

A further level of optical confinement is produced by atomic-scale metallic protrusions (adatoms) from the bulk metal that act as lightning rods to enhance optical fields even more,^4,5^ creating transient plasmonic picocavities with <1 nm^3^ effective volumes.^6−8^ Due to the small spatial extent of this enhanced field, light–matter interactions are meaningfully modified only for a single nearby molecule. Contrary to ensemble measurements that average the local environments of many molecules, this single molecule scattering fingerprint possesses narrower spectral widths and noticeable spectral wandering that depends on spectral integration time.^6,9^ While the formation of picocavities is known to depend on nearby chemical moieties^10^ and on incident optical power,^6^ the role of light in the protrusion of atomic features from the bulk metal, and their location, is currently unclear.

SERS spectra from picocavities have previously been shown to provide information about the single molecules which interact with their enhanced fields.^11,12^ Here, we demonstrate that these spectra also contain information on the local nanocavity field strength and, therefore, the picocavity location. This localization of the picocavity is achieved by comparing SERS captured simultaneously using multiple copolarized incident wavelengths (λ_1_ 633 nm, λ_2_ 785 nm) from an individual nanostructure known as a nanoparticle-on-mirror (NPoM, Figure 1a). Previously, SERS scattering from picocavities on the surfaces of silver nanoshells (a different plasmonic construct formed from silver-coated SiO_2_ spheres) was indeed shown to differ at different scattering wavelengths.^13,14^ We show here that this is in fact a direct probe of the different local field profiles formed within the nanocavity at different excitation wavelengths, thus accessing the spatial distribution of picocavity formation. By combining information from over 2500 transient events automatically identified from millions of SERS spectra, a model that picocavities are uniformly generated across the metal surface is not supported. Instead, our data suggests that picocavities form faster at higher intensity locations, consistent with an optical suppression of the energy barrier for adatom formation. We note that picocavities are never observed at low laser powers and are thus not present after self-assembly of NPoMs (likely adatoms and step edges are annealed out by the strong van-der-Waals attraction). Our data also supports prior simulations^15^ suggesting that picocavities show spectral resonances in local enhancement, dependent on how far the adatom protrudes from the facet. Dynamic changes in relative SERS intensity from different Raman lasers thus directly reflects atomic-scale restructuring of the metal surface as a single picocavity atom moves subangstrom distances.

**Figure 1:**
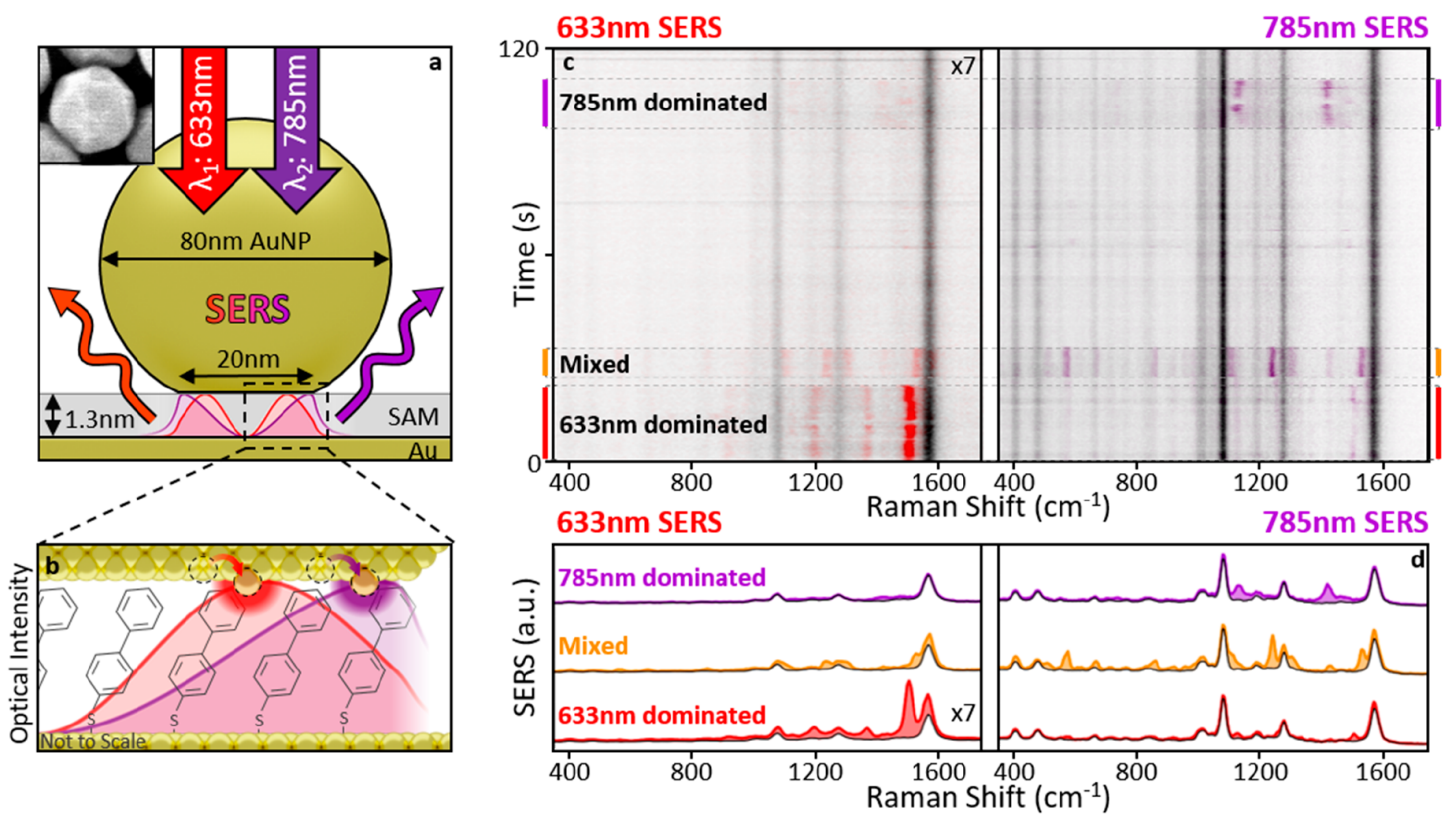
Wavelength-dependent picocavity scattering. (a) The nanoparticle-on-mirror (NPoM) construct strongly confines the optical field between a gold nanoparticle (AuNP) and gold mirror. (inset) SEM shows example AuNP facets. (b) AuNP–mirror gap defined by close-packed self-assembled monolayer of biphenyl-4-thiol. Optical modes excited at 633 and 785 nm (shown for normal incidence) possess different intensity distributions due to combinations of plasmonic modes excited. Atomic-scale protrusions from the gold facets (picocavities) further enhance fields within 1 nm^3^ effective volumes. (c, d) Surface enhanced Raman scattering (SERS) spectra from a NPoM taken consecutively over 120 s at both 633 and 785 nm scattering wavelengths (100 and 300 μW, respectively). SERS at 633 nm multiplied by 7 for visibility. Spectra corrected for instrument response function. Transient scattering features (colored) are generated by picocavities due to the strong field and gradients over a single molecule. Transient intensity varies depending on the location of the picocavity under the AuNP and can be stronger (relative to nanocavity SERS, black) in 633 or 785 nm spectra (denoted “633 nm/785 nm dominated” respectively).

## Results and Discussion

The NPoM constructs are each formed from a Au nanoparticle (AuNP) separated from a locally flat Au surface^1,16^ (Figure 1a) using robust molecular spacers,^17,18^ 2D monolayers,^19^ colloidal quantum dots,^20^ or DNA origami.^21^ These can be produced en masse by random AuNP deposition from solution onto a Au surface prefunctionalized with the spacer material. Here, we use a close-packed self-assembled monolayer (SAM) of biphenyl-4-thiol^22^ (BPT, Figure 1b). Each NPoM contains a single plasmonic hotpot (nanocavity) within the Au-mirror gap which provides a consistent and stable SERS response from the SAM (Figures 1c [black] and S1). The resonant frequencies and coupling of the plasmonic modes supported by this nanocavity depend on the gap spacing, nanoparticle size, and the refractive index of the spacer material, but the largest variation in NPoMs of the same type arise from differing AuNP size and faceting^23^ stemming from their intrinsic crystalline structure.^24^ The typical AuNP geometry adopted for simulations is an 80 nm diameter sphere truncated to create a 10 nm radius circular facet (Figure 1a, b). Each plasmonic mode possesses a different spatial profile of optical field in the gap,^25^ which linearly combine to form a total field profile dependent on the excitation angle and wavelength (Figure S2). Depending on the incident laser power and SAM molecules used, occasional transient SERS events are observed due to the formation of picocavities. For the single molecule interacting with this atomic-scale plasmonic feature, SERS is further amplified by its extra enhancement of the local field *E*_nano_ in the NPoM gap (*E*/*E*_nano_)^4^ ∼ 10^1^–10^4^ while also inducing a strong field gradient over the molecule leading to broken selection rules.^26^ This leads to the characteristic appearance of nominally dark SERS modes in picocavity spectra and allows for the single molecule SERS to be spectrally isolated from the persistent signal from all other molecules. This transient SERS is used to probe the underlying nanocavity field profiles through simultaneous continuous wave excitation using two wavelengths (λ_1_ = 633 nm, λ_2_ = 785 nm). These wavelengths are sufficiently separated in energy to allow the resulting SERS spectra to be fully resolved at the same time (termed 2-λ SERS) (Figure 1c).

During 2-λ picocavity events, modified SERS scattering is observed from both pump wavelengths (Figure 1c). However, the intensity of this transient scattering (internally normalized by the respective persistent nanocavity SERS lines) is different and varies strongly between events even from the same NPoM (Figure 1c, d). This directly follows from the underlying difference in the enhanced local nanocavity intensity for each wavelength at the location of picocavity formation.

**Figure 2:**
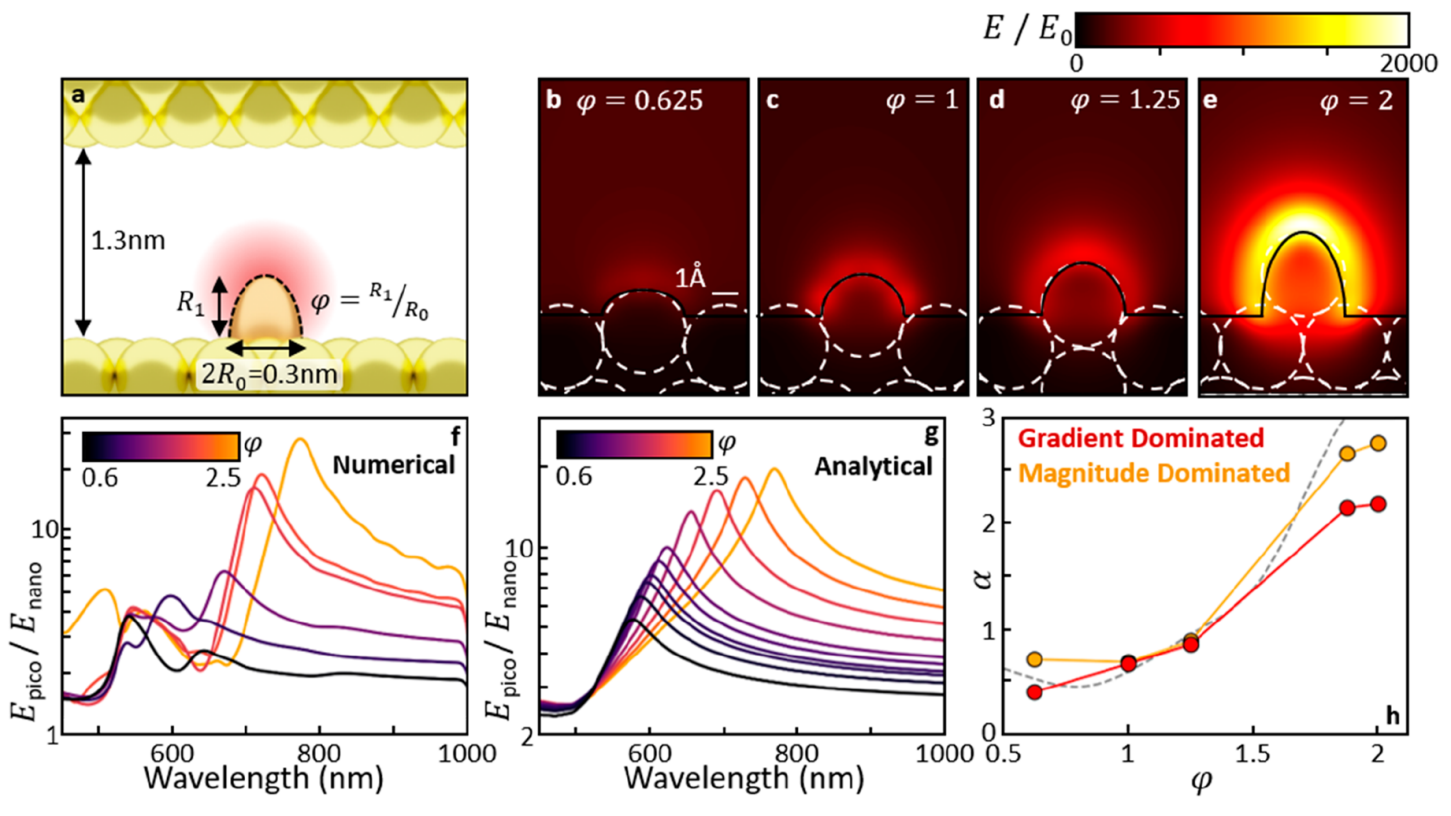
Numerical simulations of picocavity optical response. (a) Picocavity protrusion modeled as a half-ellipsoid above the flat gold mirror, positioned below the center of the AuNP. The modeled AuNP is an 80 nm diameter gold sphere truncated to give a 10 nm radius circular facet. (b–e) The local field enhancement increases with ellipsoid aspect ratio (φ). Each field is shown at the wavelength of maximum enhancement. (f) Field enhancement, normalized by the nanocavity field, displays complex resonances. (g) Analytic model of dominant resonance using hemiellipsoid in uniform field. (h) Ratios of effective field enhancement between 785 and 633 nm (α) extracted at a point 0.05 nm above the protrusion tip in FDTD, in the limit for response dominated by SERS or gradient-SERS. The dashed line is an analytical approximation of the former.

In addition, any spectral-dependence on the local field enhancement of the picocavity will also modify the ratio of SERS from the two wavelengths. To explore the near-field response of these atomic-scale features, full finite-difference time-domain (FDTD) simulations are applied to the truncated sphere NPoM model. A protrusion is situated on the mirror directly below the center of the nanoparticle, simulated as a half ellipsoid with ratio φ between the two semiaxes (Figure 2a and Supporting Information section Picocavity Modeling). A protrusion down from the nanoparticle provides the same results due to the uniform surface-normal fields across the NPoM gap. This geometry for the classical calculation thus avoids the complexity of subatomic scale crevices below a sphere that can dominate the final response but which washes out in quantum simulations. Such classical simulations of atomic scale features have held up well in a previous comparison to full quantum calculations.^4^ This also matches recent calculations treating the protrusion as a nanorod.^15^ As φ increases, the resulting protrusion increases in sharpness (tip curvature ∝ φ for an ellipsoid), and the magnitude of the surrounding near-field increases accordingly (Figure 2b–e).

A key result observed here is that the near-field magnitude taken 0.05 nm above the protrusion (*E*_pico_, normalized by the unmodified nanocavity field *E*_nano_) displays resonant enhancements that tune in both magnitude and wavelength with φ (Figure 2f). While the full response contains multiple modes, the dominant resonance can be described well by the quasi-static field above an ellipsoidal metallic particle in contact with a metallic half space within a uniform field (Figure 2g and Supporting Information section Picocavity Modeling). Both the near-field enhancement and field gradient need to be considered when predicting local picocavity enhancements of single molecule SERS. To compare our model to experimental observations, the extracted increase in effective near-field strength at 785 nm relative to 633 nm (α, recorded 0.05 nm above the tip of the protrusion) is found to be similar if either field magnitude or field gradient effects dominate (Figure 2h and Supporting Information section Estimating α). Both ratios follow the same trend, from below unity for φ ≲ 1.25 increasing to 2–3 for the maximum possible φ = 2.

**Figure 3:**
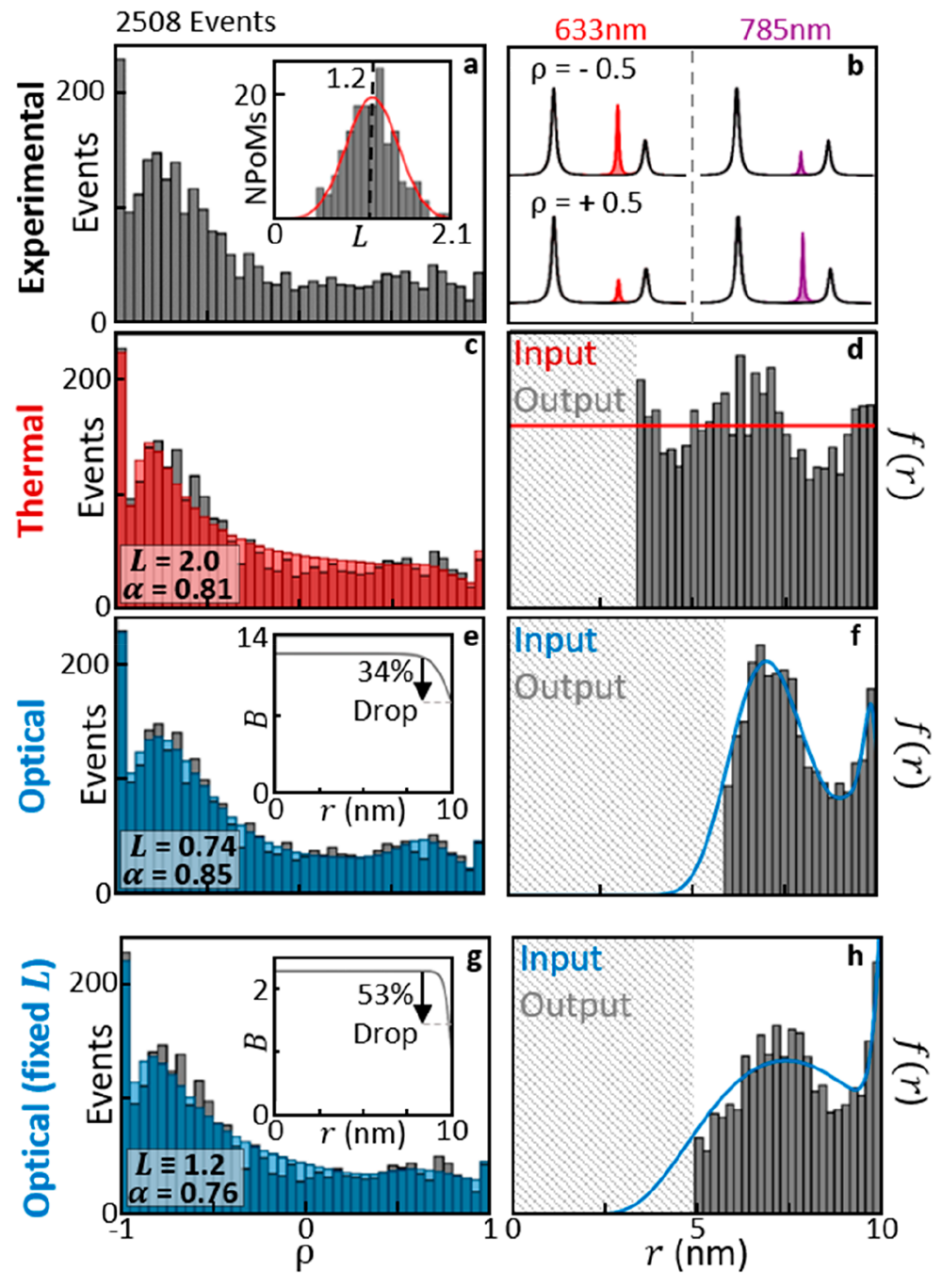
Picocavity scattering ratios over many events. (a) 633–785 nm picocavity scattering ratios *R*, normalized by nanocavity SERS, for 2508 picocavity events from 168 NPoMs. Using ρ = (1 – *R*)/(1 + *R*) these are mapped onto the {−1, 1} range. (inset) Nanocavity SERS allows estimation of parameter *L*. (b) Schematic spectra showing how sign of ρ shows which wavelength gives stronger picocavity SERS. (c) Histogram (red) optimized to match experimental results (gray), using thermally driven picocavity model with energy barrier uniform everywhere under the AuNP facet (thermal model). (d) Model-inverted data gives experimental radial adatom positions across the facet, showing large deviation from uniform formation probability (red). (e) Optimized histogram for model with energy barrier reduced by local optical intensity (optical model). (inset) The model energy barrier drops near facet edge where surface atom coordination number is lower. (f) The optical model shows better agreement between input formation probability (blue) and inverted adatom positions. (g, h) The optical model also recreates experimental histogram well for fixed *L*. Optimized barrier energy falls by ∼50% at the facet edge (inset).

To investigate picocavity scattering ratios over many events, 2-λ SERS spectra are collected individually from 561 NPoMs with SERS spectra taken every 200 ms for a period of 2 min. This results in a total of >670 000 spectra considering both scattering wavelengths. These are manually filtered to the 168 NPoMs displaying clear BPT SERS with picocavity events (30% of NPoMs at these incident laser wavelengths and powers). The relative intensity of these spectra are corrected by the spectrally dependent instrument response of the experimental system (Figure S4), and robust algorithms to remove background and persistent SERS emission separate the transient picocavity scattering from the nanocavity contributions (Supporting Information section Extracting Transient SERS spectra). This results in a data set of 2508 detected picocavity events (Supporting Information section Defining Picocavity Events). The intensity of SERS spectra collected at each wavelength are dependent on both the incident optical powers (here 633 nm: 100 μW and 785 nm: 300 μW on sample) and the optical in/out coupling efficiencies of the NPoM cavity at each wavelength.^27,28^ A spectrally dependent scaling is thus applied to correct for in/out coupling effects using the nanocavity spectra as an internal reference (Supporting Information section Defining Picocavity Events). After this normalization, the 633:785 nm picocavity SERS ratio (*R*) is extracted for each event and remapped onto the finite range {−1, 1} using the metric ρ = (1 – *R*)/(1 + *R*) (Figure 3a). Here, ρ = 0 represents equal normalized scattering at both wavelengths while ρ < 0 (ρ > 0) represents greater scattering at 633 nm (785 nm) respectively (Figure 3b). Experimentally observed values of ρ extend over the entire available range with 72% of events showing more dominant 633 nm scattering (Figure 3a, b).

To numerically recreate this experimental distribution requires the combination of two independent models. First, a model is needed to describe the value of ρ expected for a picocavity at any given position within the NPoM gap. Second, the probability density function (PDF) for the generation of picocavities within the gap must also be defined. These will be discussed separately. The normalized SERS ratio *R* from a picocavity at position *x̲* under the nanoparticle for wavelengths {λ_1_, λ_2_} can be calculated from the field profiles *E*(*x̲*, λ) = *A*_λ_ψ(*x̲*, λ) extracted from FDTD simulations. The cavity and the positions where adatoms can form are defined by the 2D surface *S* of the nanoparticle facet. Vector *x̲* is therefore a given 2D location on *S* where we consider the possible formation of a picocavity. Note this also describes picocavities forming on the mirror surface directly below the nanoparticle facet.^10^ Any chemical interactions between the adatom and molecule could influence the final picocavity SERS intensity, but as this is not optically dependent, it is not included here (though our model is extendable to include this if desired). It is important that the laser photon energies are detuned far below the electronic absorption of the BPT molecule so that the SERS is not electronically resonant.

The SERS ratio depends only on the local intensity at each wavelength and the ratio of picocavity near-field enhancements at λ_2_ vs λ_1_ (α), giving (Supporting Information section Defining Picocavity Events)
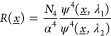
1where *N*_4_ ensures normalization of the transient signal to the nanocavity SERS, defined using
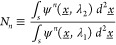
2The parameter α is free to optimize and will be compared below to estimates from the ellipsoidal picocavity FDTD model (Figure 2). This means that this expression is affected only by numerical models of the nanocavity field distributions and does not require an FDTD (or other numerical) model of the picocavity structure itself. Experimentally, some signal from weak SERS events (comparable to the experimental noise) at either wavelength will be missed and/or be excised as part of the nanocavity SERS background. To account for this, the theory model passes the picocavity signal through a suppressing function *s*(*I*; *I*_0_) = max(*I* – *I*_0_, 0) that similarly clips off low signals. The simple form for this function helpfully reduces the total number of model parameters, giving
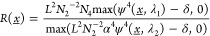
3where δ parametrizes the experimental noise and *L* = *N*_2_(*A*_λ_2__/*A*_λ_1__)^2^ gives the ratio of optical powers coupled into the NPoM cavity, which alters the relative influence of noise on the SERS from each wavelength postnormalization (Supporting Information section Model Derivation). This model simplifies to eq 1 in the case δ = 0 and becomes undefined for locations where SERS at both wavelengths is too weak to be detected above the noise.

This general model can be applied to any nanocavity field distribution. Here, it is applied to the truncated sphere NPoM model (Figure 1a) which provides a reasonable approximation to the range of faceted shapes observed.^27^ We fit a single value of α for all events, thus averaging over adatom protrusion extent (since picocavities are metastable suggesting they have a preferred position). Due to the symmetry of this geometry, the field distributions are described by separable terms depending on radial distance (*r*) from the facet center and the polar angle (ϕ). As polar angle dependence is not wavelength dependent, it can be ignored and field strengths expressed as the radially symmetric *E*(*r*, λ) = *A*_λ_ψ(*r*, λ). Depending on the excitation angle in FDTD simulations, the excited nanocavity fields either display a minimum at the facet center (near-normal excitation) or a maximum (high angle excitation). We find that the former (Figure 1a; normal incidence) much better describes the data than the latter (Figure S17).

To simulate the distribution of *R* (or ρ), the formation of picocavities within a ring d*r* on the facet at radius *r* is defined with PDF = *f*(*r*)*r* d*r* and used to explore different formation models. For a given model with *f*(*r*) optimized to best match the experimental histogram (Figure 3a), the experimental data can be inverted to give adatom radial positions within the model, that are then compared directly to this input *f*(*r*) (note this inversion is only possible when the resulting ρ(*r*) is monotonic in the range |ρ| < 1). The energy barrier that must be overcome to generate a picocavity is denoted as *B*(*r*), with *f*(*r*) ∝ exp{−*B*(*r*)/*k*_B_*T*}. Including a sigmoidal decay at the facet edge, *B*(*r*) then accounts for the reduced coordination number of surface atoms at edges. Our simplest thermal model assumes both a constant energy barrier (with no drop at the facet edge) and that optical illumination only drives picocavity generation though NPoM heating. Rapid thermal diffusion in gold ensures each nanoparticle is uniform in temperature and thus *f* becomes a constant. Optimizing the values of {α, *L*, δ}, this thermal model already reproduces the general shape of the experimental histogram (Figure 3c). However, re-expressing the experimental adatom positions within this model gives an undulating radial distribution in contrast to the flat input *f*(*r*) = *f* (Figure 3d). Even so, this simplest model does show an increase in experimental picocavity generation around *r* ∼ 6 nm where optical fields are largest. Further including the sigmoidal decay in *B*(*r*) increases the number of free parameters from 3 to 5, while only slightly improving agreement between model and recovered distributions (Supporting Information section Optimizing Picocavity Models).

We now consider a more direct role for local optical fields in adatom formation. As optical forces are too weak to generate adatoms directly (Supporting Information section Optical Forces), we still consider picocavities to be thermally driven (since they arrive stochastically in time) but with their effective energy barrier reduced by the presence of local field intensity *I*(*r*). Without a definitive mechanism for this barrier suppression (as the picocavity generation mechanism is yet unknown), we model this with the linear suppression *B*^OM^(*r*) = *B*(*r*)/[*I*(*r*) + *c*]. In the low power limit *I*(*r*) ≪ *c* of this optical model, this returns to the thermal model with constant *c* keeping the effective barrier finite. We consider here the opposite limit *I*(*r*) ≫ *c*, which minimizes model parameters. As the total optical power (combining both pump wavelengths) increases, this local barrier reduces. For the low powers here, their intensities can be simply summed to give
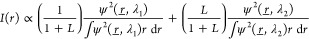
4weighted by the ratio of total optical powers (*L*) coupled into the nanocavity at each wavelength discussed previously. As *f*(*r*) is expressed in terms of the ratio of *B*(*r*) to *I*(*r*), the latter is normalized to max[*I*(*r*)] ≡ 1 without loss of generality. Because of this, only the shape of *B*(*r*) is required for the model. This model captures various possible physical mechanisms, such the provision of nonthermal energy through a momentum transfer to a surface atom from electronic Raman scattering within the bulk gold (additional weightings discussed in Supporting Information section Optimizing Picocavity Models). Forcing *B*(*r*) to be constant, the system optimizes back to the thermal model (Supporting Information section Optimizing Picocavity Models). Using the sigmoidal form of *B*(*r*) now gives much better agreement between the inverted experimental adatom positions and the input *f*(*r*) for this optical model (Figure 3e, f), with now six free parameters. This *f*(*r*) has a peak at *r* ≃ 6 nm where the nanocavity intensity is strongest, along with a second increase in adatom formation probability near the facet edge due to the drop in barrier energy *B*(*r*).

Allowing the fitting parameters full freedom optimizes the thermal model at *L* = 2.0 (Figure 3c), compared to the optical model at *L* = 0.74 (Figure 3e), contradicting which wavelength couples in more light. Using the nanocavity SERS emission and FDTD simulations to correct for out-coupling efficiency, this parameter can be experimentally determined (Figure 3a inset, Supporting Information section Fixing L) as normally distributed around *L* = 1.2, with the distribution width resulting from variations in nanoparticle shape and size between NPoM constructs. Fixing *L*, a reoptimized thermal model is unable to replicate the experimental histogram and can no longer even recreate the full range of ρ from −1 to 1 (Supporting Information section Optimizing Picocavity Models), even with sigmoidal *B*(*r*).

The optical model with sigmoidal *B*(*r*) now has five free parameters and still replicates the experimental histogram well (Figure 3g, h). The resulting α = 0.76 indicates 30% stronger picocavity near-field enhancement at 633 nm compared to 785 nm. This is well within the reasonable range given by numerical simulations (Figure 2) and suggests aspect ratios ϕ ∼ 1 as perhaps expected (hemispherical protrusion on average). This model implies that the picocavities which are dominating at 785 nm SERS form near the facet edge (Figure S29) due to the 50% drop in *B*(*r*) over a characteristic sigmoid width of 0.2 nm which is around the size of a gold atom (Figure 3g, inset). Effects from less decrease in energy barrier, or the noise parameter δ, are shown in Figures S29–30. Overall, our data strongly excludes thermal heating as the role of light in generating picocavities and supports alternative models based on light-induced extraction.

**Figure 4:**
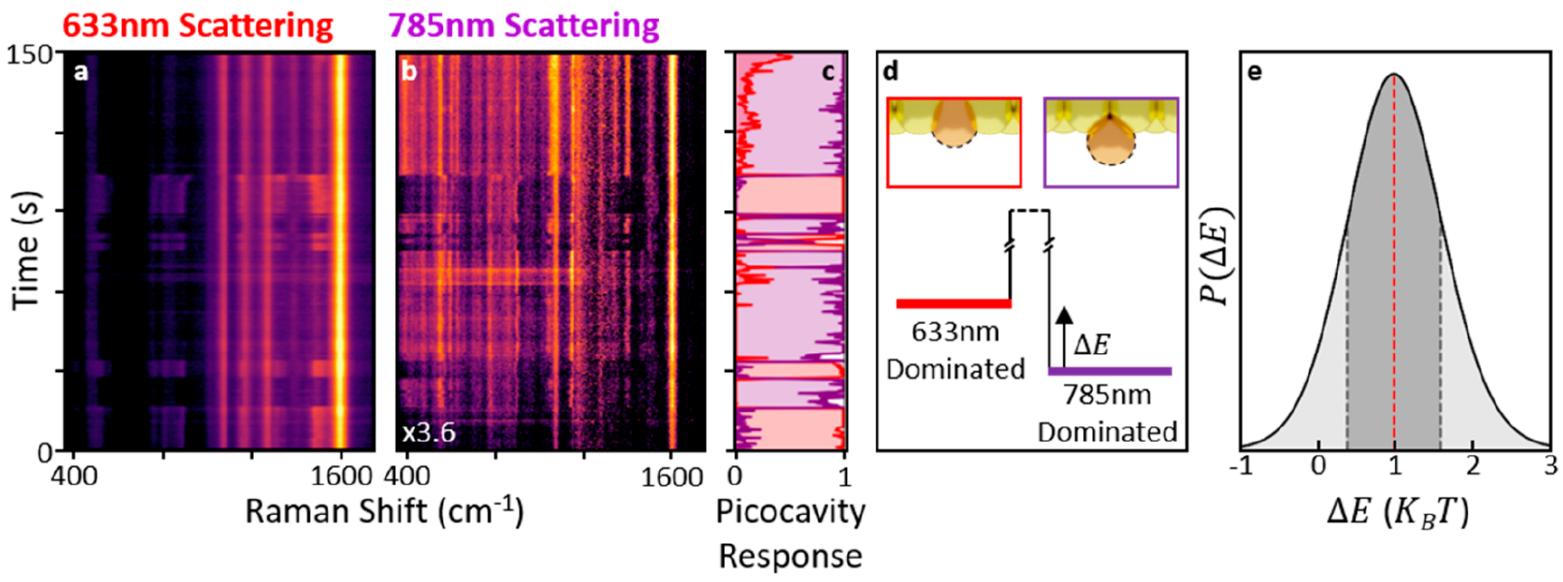
Switching in dominant scattering wavelength. (a, b) SERS showing repeated switching between dominance in each wavelength. (c) Characteristic spectra from 633 (50) and 785 nm (280 μW) SERS provide a direct metric for the picocavity components, showing that switching is strongly anticorrelated between them. (d) Model of two metastable states with energy difference Δ*E* separated by an unknown energy barrier. The schematics show their possible origin as different height adatom protrusions. (e) Observed lifetimes in each metastable state yield a probability density function for Δ*E*. The maximum likelihood is 1.0*k*_B_*T*, and the shaded area indicates 67% probability.

Very occasionally, a picocavity event is observed in which the SERS intensity repeatedly switches between dominance in each wavelength (Figure 4a). This example, taken with 50 μW 633 nm and 380 μW 785 nm, is drawn from a smaller data set for which the model parameters cannot be fully optimized. In 633 and 785 nm SERS, the spectra can be reconstructed at each time step as a linear combination of 2 or 3 characteristic spectra, respectively. This allows the weighting of the picocavity components to be plotted over time and clearly shows anticorrelated repeatable changes in system state (Figure 4c). From the conclusions above, this switching behavior can only be described by reversible adatom movement. For movement laterally under the nanoparticle facet, this would require rapid reversible changes in position over >2 nm distances which we consider improbable. Instead, this can be understood as reversible facet rearrangement leading to repeated changes of ±0.1 nm in adatom protrusion distance. Considering the lifetime of each metastable state before switching as controlled by some unknown energy barrier (Figure 4d) gives exponential PDFs for each state lifetime. Comparing the ratio of lifetimes in each state, the most likely energy difference between these two metastable states of the system is extracted as (1.0 ± 0.6) *k*_B_*T* (Figure 4e, Supporting Information section Extracting Metastable State Energy Difference). The breadth of this PDF is a direct result of the small number of switches observed. Quantitative interpretation of 2-λ SERS data in this way thus gives direct insights into metastable states on subatomic length-scales that can be extended in future work.

## Conclusion

In conclusion, wavelength-dependent picocavity scattering is dependent on both the intrinsic resonant enhancement from the atomic scale structure and the local nanocavity field at the picocavity location. Simultaneous measurements at two (or more) pump wavelengths quantifies the SERS ratios and gives histograms confirming that picocavities can form at many different locations on the gold facets. We propose a model for this SERS ratio, which cannot reproduce the experimental data if adatoms are equally likely to be generated at any position within the NPoM gap. Instead, the model best matches the data when picocavities are generated more frequently at regions of higher optical intensity. This is modeled by an optical suppression of the formation energy barrier, but as insight into the picocavity generation mechanism develops, other functional forms also giving more picocavity formation where intensities are higher should also fit the model well.

Our model is optimized using a large distribution of experimental transient events. This allows differences between individual events to be averaged over, such as differences in AuNP crystal shape, size, and differences in the atomistic structure around the picocavity. The model is kept as simple as possible to minimize free parameters but still describes the experimental data well. Currently we ignore the possibility of different picocavity formation energy barriers at the mirror and nanoparticle surfaces,^10^ which are here combined into a single effective barrier. Dynamic changes in picocavity scattering ratios during a transient SERS events represent a rich source of information on the picocavity structure that is averaged over in this work. Using such dynamic data would give new sources of insight for the picocavity structure which would benefit from nonclassical simulations of the gold structure around a picocavity and the resulting fields. To apply the model successfully to individual events, some of the parameter averaging could be removed: for example, AuNPs with consistent crystal shape could beneficially be used in future studies, allowing the nanocavity FDTD simulations to more accurately represent the gap and facet of each NPoM gap.

Although the picocavity is discussed here in terms of an adatom feature, our model does not require a calculated picocavity FDTD field as input. Instead, the model only requires that the picocavity enhances local SERS and that it is able to enhance one scattering wavelength over another. This means that, as atomistic models for the gold around a picocavity protrusion become more refined (or if an alternative explanation for the field confinement of a picocavity is developed), this model remains valid. The final optimized model developed here supports greater SERS enhancement at 633 than at 785 nm, experimentally supporting the presented concept^15^ that picocavity enhancement is spectrally resonant.

While the precise theoretical mechanism behind picocavity generation and the role of optical fields remains to be formulated in a quantum description, our data and model provide key insights toward understanding the formation and stability of these atomic scale plasmonic constructs.

## Methods

### Sample Preparation

An atomically smooth silicon wafer was cleaned using Decon 90, water, ethanol, and isopropanol. A 100 nm layer of Au was deposited on the wafer using a Lesker E-beam evaporator. Small (5 mm × 5 mm) silicon pieces were glued to the Au layer using Epo-Tek 377 epoxy and allowed to cure at 150 °C for 2 h before being gradually cooled to room temperature. When peeled away, each silicon piece removes the gold from the larger wafer exposing a flat clean gold surface on the piece. This was submerged in a 200 proof ethanol solution of 0.1 mM BPT and incubated overnight. After incubation, the samples were rinsed using ethanol and 20 μL of an 80 nm Au nanoparticle colloidal suspension (BBI Solutions, citrate stabilized, OD1) was deposited on the now hydrophobic sample surface. After 20 s the droplet was rinsed off using DI water and blown dry with nitrogen.

### Data Collection

A home-built Raman setup was constructed allowing two volume Bragg grating filtered diode lasers (632.8 and 785 nm) to be coupled in simultaneously using both a 650 nm dichroic and a 10/90 beam splitter. A simplified diagram is shown in Figure S32. Using a motorized stage and an imaging camera, NPoM constructs were automatically identified for laser irradiation allowing for a large number of particles to be characterized to obtain statistics. The NPoM scattered light was sent through four notch filters (2 × 633 nm and 2 × 785 nm) and collected using an Andor Newton 970 BVF EMCCD coupled to a Triax 320 spectrometer with a 150l/mm grating to allow for sufficient spectral range to collect both 633 and 785 nm SERS simultaneously. The instrument response function was characterized using the calibrated emission spectrum from a halogen lamp and a white light scattering reference.

## Supporting Information Available

All figures and notes referenced in the manuscript, including additional details on FDTD simulations and experimental data analysis, a full derivation of the presented analytical model, and a comparison of differing models optimized to the experimental results (PDF)
