# Supplementary material for: Locating Single-Atom Optical Picocavities Using Wavelength-Multiplexed Raman Scattering
Source: ACS Photonics. 2021 Oct 4;8(10):2868–75. doi: 10.1021/acsphotonics.1c01100 (PMC8532146; doi:10.1021/acsphotonics.1c01100)
Supplement: Supplementary file 1 — ph1c01100_si_001.pdf [file ph1c01100_si_001.pdf]

Locating Single-Atom Optical Picocavities using Wavelength-Multiplexed Raman Scattering

Supporting Information

Jack Griffiths[1†], Bart de Nijs[1†*], Rohit Chikkaraddy[1], Jeremy J Baumberg[1*]

[1] NanoPhotonics Centre, Cavendish Laboratory, J J Thomson Avenue, University of Cambridge, CB3 0HE, UK
[*] corresponding authors. email: jjb12@cam.ac.uk, bd355@cam.ac.uk

Pages: 31
Figures: 32

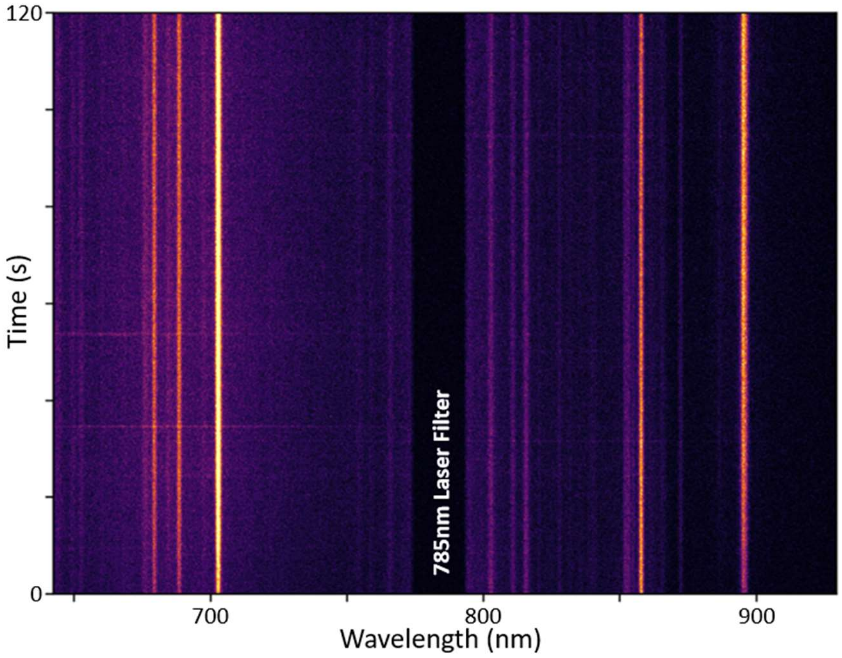

Figure S1 | Stable 2-λ SERS spectra. Example series of SERS spectra taken of a BPT NPoM under simultaneous 633 nm (100 µW) and 785 nm (300 µW) irradiation, showing stable emission with no transient SERS peaks. These spectra are raw and not corrected by the experimental instrument response function.

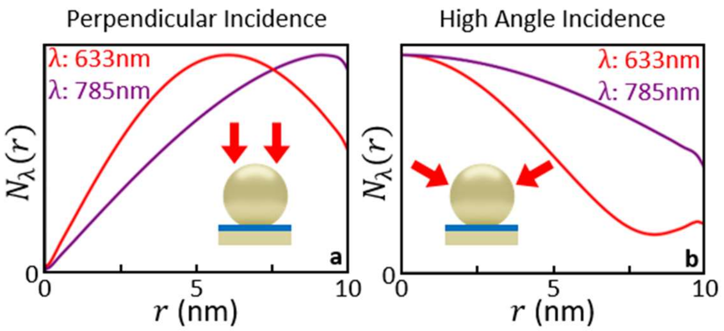

Figure S2 | Nanocavity Plasmonic Modes. Due to symmetry, only the dependence on radial distance r from the AuNP facet centre for the calculated NPoM field profiles is relevant here. These profiles from FDTD simulations, differ depending on angle of incidence [26]. **a,** Near normal incidence shows a node in the centre whilst **b,** high angle excitation results in a maximum at the centre of the cavity.

Picocavity Modelling

Numerical Simulations:

The near-field optical response of the NPoM and picocavity structures are obtained from 3D full-wave Finite Difference Time Domain (FDTD) simulations using *Lumerical FDTD*. The BPT molecular spacer is represented with a 1.3 nm dielectric layer of refractive index 1.45. The AuNP is modelled as an 80 nm

diameter sphere truncated to form a 10nm radius circular facet. The gold permittivity was taken from Johnson and Christy (DOI: 10.1103/PhysRevB.6.4370). Small mesh refinements of 2 nm, 0.2 nm, and 0.02 nm are used around the AuNP, spacer and picocavity protrusion respectively. To calculate the optical response, the NPoM is illuminated with a broadband plane wave with polarization perpendicular or normal to the mirror to extract thse near-field enhancements for the nanocavity as a function of wavelength. The picocavity is modelled using an additional half-ellipsoid protruding out of the mirror at its centre, fixed in radius along both axes parallel to the mirror at $R_{x,y}$ = 0.16 nm (the size of an Au atom) with a perpendicular length R_z tuned as shown in Figure S3a. The picocavity near-field spectrum is extracted at a point 0.05 nm above the ellipsoid tip and normalised with the nanocavity field. In both cases, the simulations are carried out with identical sample meshing and boundary conditions.

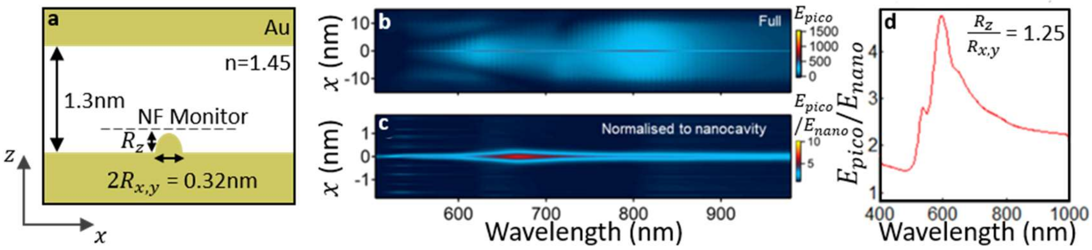

Figure S3 | Numerical Field Simulations. a, Geometry (to scale) calculated in FDTD simulations, with an ellipsoidal protrusion from the NPoM mirror. The near-field is extracted 0.05 nm above the protrusion. **b,** The near-field spectrum vs wavelength and vs lateral position from the protrusion. The protrusion has a ratio between semi-axes of 1.25. **c,** Map in (b) normalised by the field with no protrusion, showing a clear resonant enhancement at the protrusion position x=0. **d,** The enhancement spectrum extracted directly above the protrusion.

Analytical model:

The polarizability of a half-ellipsoid on a metallic surface is classically estimated under the approximation of uniform fields and quasi-static limits. The polarizability of the system in a dipole approximation is given by

$$\alpha_j = V \frac{\epsilon - \epsilon_m}{\epsilon_m + L_s L_j (\epsilon - \epsilon_m)} \quad , \quad j = \{x, y, z\} \tag{1}$$

where V is the volume of polarising half-ellipsoid, ϵ is the complex wavelength-dependent permittivity of Au (as used in the FDTD simulations) and ϵ_m is the permittivity of the medium (set by the refractive index of BPT) (doi: /10.1039/C6CS00919K). The coordinate system is defined with Cartesian coordinates $\{x, y\}$ in the plane of the metallic surface with z perpendicular. L_j are structure parameters accounting anisotropy of the ellipsoid and are subject to the condition $\sum_{j=1}^{3} L_j = 1$. The relevant polarizability here is α_z, for which

$$L_z = \frac{R_{x,y}^2}{2} \int_0^\infty \frac{ds}{(s + R_z)^2 \sqrt{(s + R_z^2)(s + R_{x,y}^2)^2}}$$

$$= \begin{cases} \dfrac{1-e^2}{e^3}(\tanh^{-1} e - e)\,, & e = \sqrt{1 - {R_{x,y}}^2/{R_z}^2} \quad \text{if } R_z > R_{x,y} \\ \dfrac{1+e^2}{e^3}(e - \tan^{-1} e)\,, & e = \sqrt{{R_z}^2/{R_{x,y}}^2 - 1} \quad \text{if } R_z < R_{x,y} \end{cases} \tag{2}$$

where R_j are the semi-axes of the ellipsoid (doi: 10.1021/jp066539m). The structure parameter L_s describes the connection of the ellipsoid to a polarising metal surface and is given by

$$L_s = 1 - \frac{0.4(\epsilon - \epsilon_m)}{\epsilon + \epsilon_m} \tag{3}$$

where the constant 0.4 is tuned to match the analytical result to the numerical simulations. We note this model matches well the simulations in [15].

Estimating α

Although a molecule undergoing SERS scattering is an extended structure, a point dipole approximation is applied here. The field magnitude at wavelength λ at the molecule position is defined as $E_{\text{pico}}(\lambda)$. Spatial gradients are denoted through a dash ie. $E'_{\text{pico}}(\lambda)$. The nanocavity field magnitude at this position in the absence of the picocavity structure is $E_{\text{nano}}(\lambda)$. Without the picocavity, the expected SERS scattering is $\propto E^4_{\text{nano}}(\lambda)$.

If the increase in field magnitude sets the SERS enhancement from a picocavity, this enhancement is given by ${R_\lambda}^4 \equiv \left(\frac{E_{\text{pico}}(\lambda)}{E_{\text{nano}}(\lambda)}\right)^4$. In this limit, $\alpha = \frac{R_{785\text{nm}}}{R_{633\text{nm}}}$.

If the non-negligible field gradient sets the SERS enhancement from a picocavity, this enhancement is given by ${R_\lambda}^3 \frac{E'_{\text{pico}}(\lambda)}{E_{\text{nano}}(\lambda)} \approx {R_\lambda}^3 {R_\lambda}'$ because $E'_{\text{nano}}(\lambda) \approx 0$. In this limit, $\alpha^4 = \left(\frac{R_{785\text{nm}}}{R_{633\text{nm}}}\right)^3 \frac{R'_{785\text{nm}}}{R'_{633\text{nm}}}$.

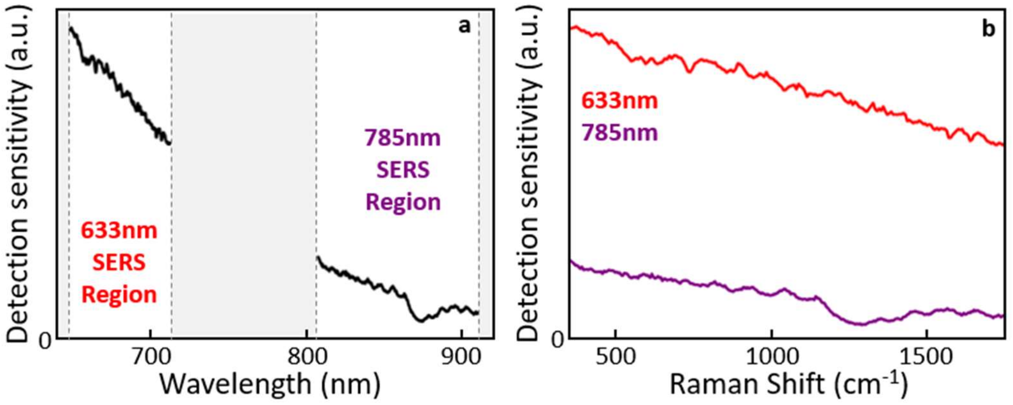

Figure S4 | Instrument Response Function. a, Experimentally determined instrument response function (normalised to 1), showing the relative optical efficiency at each wavelength. This takes into accounts all wavelength-dependent optical losses resulting from lenses, optical components such as filters and gratings, and the quantum efficiency of the CCD. **b,** Same response function as (a) shown vs Raman shift for each scattering wavelength.

Extracting Transient SERS spectra

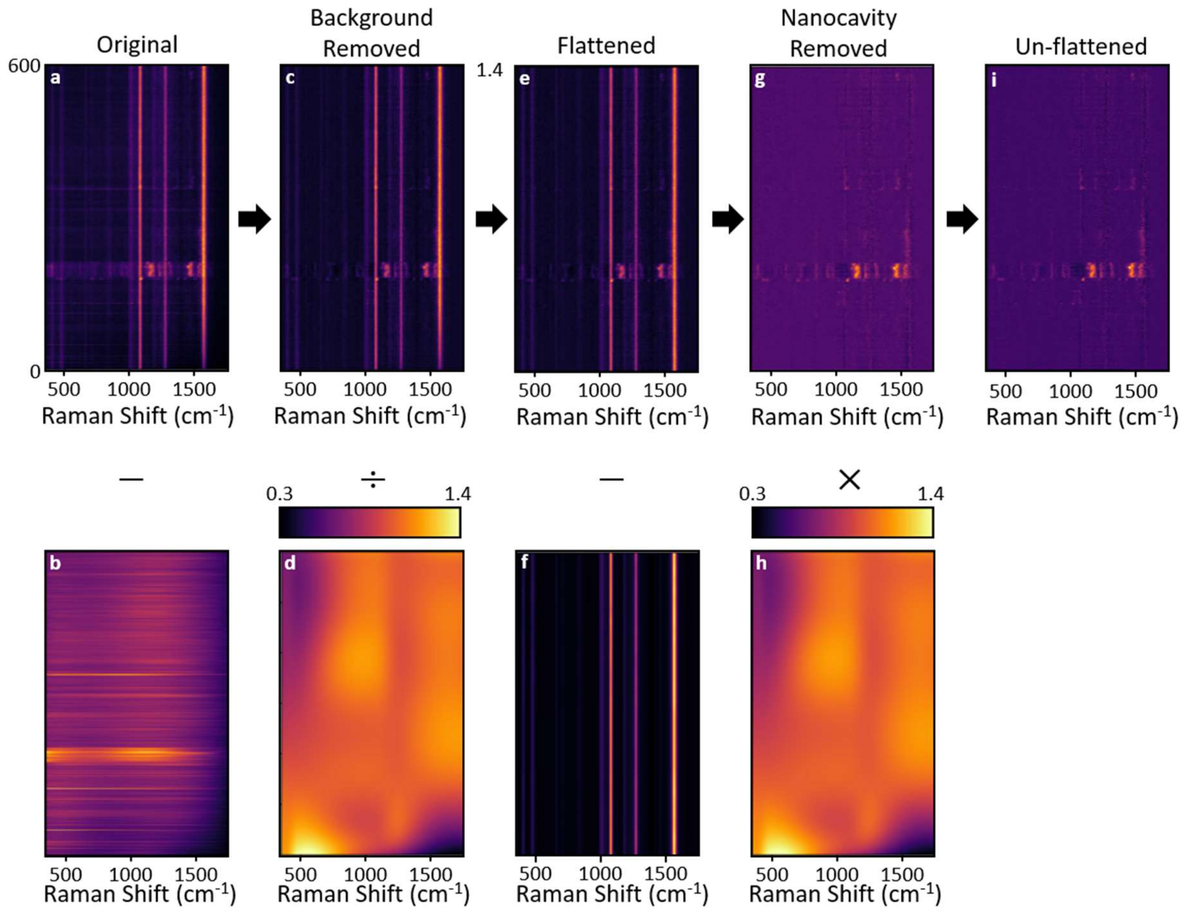

Figure S5 | Transient SERS extraction. a, Each time scan of repeated SERS spectra has **b,c** polynomial estimates for the background removed. **d,e,** The remainder is divided by a correction map to remove large-scale changes in SERS intensity. **f,g,** This makes it simpler to estimate and remove the persistent nanocavity SERS before the correction **h,i,** is reversed.

Extraction of transient *picocavity*-SERS spectra from a kinetic SERS dataset with a persistent but slowly variable *nanocavity*-SERS spectrum is performed in a multistep process (Figure S5). First, the broadband SERS background for each spectrum is estimated using a polynomial function of automatically determined order (see below) and removed. Next, any slow and largescale changes to the spectrum intensity due to, for the most part, drifts in the experimental system are corrected for by dividing through by an automatically determined correction map (see below). The suppression of these intensity drifts allows for the persistent nanocavity-SERS spectrum to be isolated from the scan and removed. With the nanocavity-SERS removed, what remains is multiplied by the correction map to reverse its application.

<u>Polynomial Background Removal</u>

Each SERS spectrum is assumed to comprise of a sum of a broadband background, narrowband SERS peaks and noise. As the SERS signal only exists over certain sections of the spectrum, masking these regions away leaves the sum of background and noise which can be fit to a smooth polynomial

function. Here, the noise is assumed to be drawn from a normal distribution. However, this methodology is extendable to any noise distribution.

The algorithm for background removal iteratively updates this mask of possible SERS peak locations. With a given background estimate in place, the new mask is defined as all spectral points that exceed this background estimate by a given dynamic threshold. This process is iterated until the peak mask and corresponding background estimate converge. This is described in detail here, but the high level process is also shown using the following flow diagram.

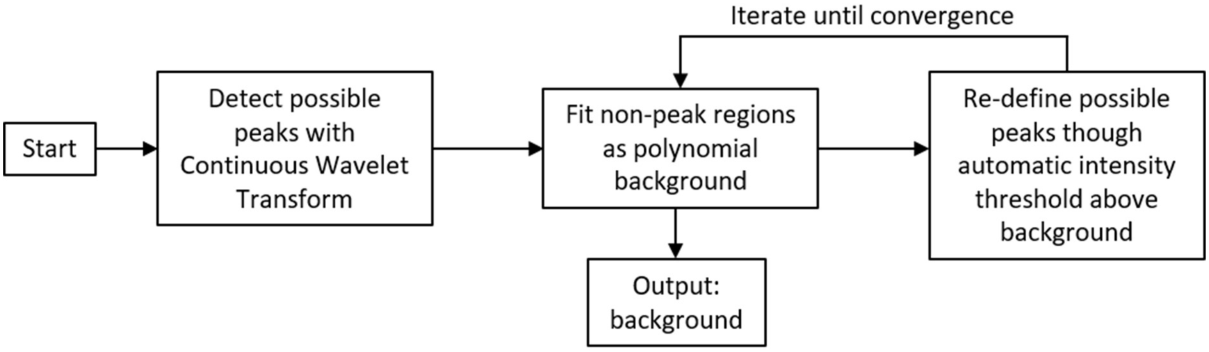

To achieve this, two sub-algorithms are defined. The first of these takes a set of points to be fit with a polynomial and automatically sets the polynomial order. The second compares similar background estimates and quantitatively ranks them to select a preference. Also described here is a method for defining the initial background estimate to be iterated and the process of that iteration.

Defining the Polynomial Order

For a given set of points (x_i, y_i) to be fit with a polynomial function, the polynomial order N must be defined. The polynomial is denoted as $P(x_i;\vartheta)$ where ϑ represents the polynomial coefficients. The residuals $R_i = y_i - P(x_i;\vartheta)$ are assumed to be normally distributed. The variance of these residuals is given by $\sigma^2 = \sum_i {R_i}^2 / \sum_i 1$. The Log-Likelihood of this model is

$$L = -\frac{1}{2}\sum_i \left[\ln(2\pi\sigma^2) + \left(\frac{R_i}{\sigma}\right)^2\right]. \tag{4}$$

The Bayesian Information criterion (BIC) is a numerical metric for model evaluation, punishing both a poor fit to the data and a large number of model parameters. When comparing models, a lower BIC can be considered a better description of the data. Here, the BIC is given as

$$\mathrm{BIC} = (N+2)\ln\left(\sum_i 1\right) - 2L. \tag{5}$$

The polynomial order that minimises this metric is taken for the background estimation. A key disadvantage of polynomial approximations is a tendency to generate oscillating solutions at higher polynomial orders. This behaviour can be suppressed by defining a maximum number of turning points permitted over the domain of $\{x_i\}$ for a polynomial order to be considered. In this work, this is set at 3 for background estimation.

Comparing Polynomial Background Estimates

A spectrum and a polynomial background estimate are comprised of discrete elements S_i and P_i respectively and the residual between them is defined as $R_i = S_i - P_i$. Considering the distribution of

all R_i, larger positive values will be dominated by values from SERS peaks. Near and below zero, this distribution should instead represent zero-centred noise. If the distribution is correctly zero-centred, the standard deviation of this noise is given by the root mean square of the negative values. This value is set as a threshold above which R_i are discarded as possibly no longer representing noise and being contaminated by SERS counts.

The remaining R_i need to be compared to a zero-centred Gaussian truncated at this threshold. This could be achieved by optimising a truncated Gaussian model with free centre and standard deviation parameters and consulting the optimised centre position. Instead, to make this method more generalizable to non –Gaussian noise distributions, two models are optimised and compared. These are a truncated Gaussian fixed to be zero-centred and a second free to translate. The second model could be increased further is complexity with the addition of a skew term but this is not done here. The log-likelihoods of the zero-centre and free-centre models are defined by L_G and $L_F \geq L_G$ respectively. Note that if the residuals perfect describe a zero-centre Gaussian, $L_F = L_G$. We define a metric

$$M \equiv \frac{2}{1 + e^{L_F - L_S}} - 1, \quad 0 \leq M \leq 1 \tag{6}$$

that approaches zero for polynomial backgrounds that provide Gaussian distributed noise residuals. This is a sensitive metric, only significantly below unity in cases where the distribution is very well described by a zero-centre Gaussian. When comparing similar estimates, the estimate with the lowest metric score can be taken (Figure S6).

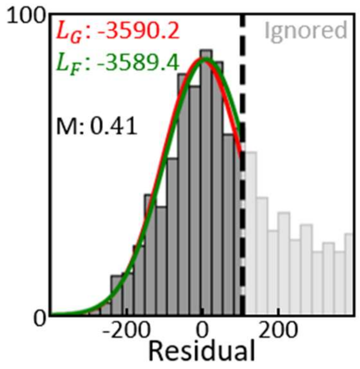

Figure S6 | Example background metric. Example residual histogram for a SERS spectrum with polynomial background estimate removed. Near and below zero, this distribution is near Gaussian. Above a threshold, the distribution is discarded as no longer describing the noise. Optimising normal distributions to this data with centres fixed at zero and free to optimise provides metric M for how well this noise is represented by a zero-centred Gaussian.

Defining an Initial Background Estimate

For any iterative method, an initial background estimate must be defined. Here, this could be a simple low order polynomial fit to the entire spectrum. A more robust but computationally costly estimate attempts to ignore possible SERS peaks by detecting them using a continuous wavelet transform. The Ricker wavelet (Figure S7a) is defined as

$$\varphi(x; \mu, \sigma) \propto \frac{1}{\sigma}\left(1 - \left(\frac{x - \mu}{\sigma}\right)^2\right) e^{-\frac{(x-\mu)^2}{2\sigma^2}}. \tag{7}$$

As this is related to the second derivative of a Gaussian function, the overlap integral

$$W(\mu,\sigma) = \int_{-\infty}^{\infty} x(t)\ \varphi(t;\mu,\sigma)\, dt \tag{8}$$

is large when the function $x(t)$ locally approximates a peak-like structure of scale σ at $t = \mu$. By integrating over all wavelet centres

$$P(\sigma) = \int \left(\frac{W(\mu,\sigma)}{\sigma}\right)^2 d\mu \tag{9}$$

is maximised when σ matches the characteristic scale of peak-like structures in $x(t)$ (Figure S7b). This can be applied to a SERS spectrum to estimate the characteristic peak and detect possible peak positions as maxima in $W(\mu,\sigma)$ at that characteristic width. For efficiency, this can be applied to a scan of spectra rather than each spectrum individually.

To prevent the possible domination of noise in $P(\sigma)$, an initial peak width estimate is defined (here 15cm^{-1}). The spectra are smoothed using a quadratic order Savitzky-Golay filter of window size 2σ to suppress noise while retaining pertinent spectral features. By maximising $P(\sigma)$ on these smoothed spectra, a new estimate for σ is defined. This is repeated until convergence. With this optimised width, possible peak positions are given by positive local maxima of function $W(\mu,\sigma)$ with respect to μ. Regions within 2σ of possible peak positions are masked and the remainder fit to a polynomial. Possible peaks below a given threshold can be discarded so that only truly resolved possible peaks are masked. Different thresholds for discarding possible peaks are tested and the resulting background with the lowest metric M is taken as the initial estimate (Figure S7c).

Iterating the Background Estimate

The new mask of possible SERS peak locations is defined as all spectrum elements exceeding the current background estimate by a given threshold. This threshold is scanned and the resulting background estimate that provides the lowest metric M is taken as the updated background estimate (Figure S7d).

This process is iterated until convergence (Figure S7e).

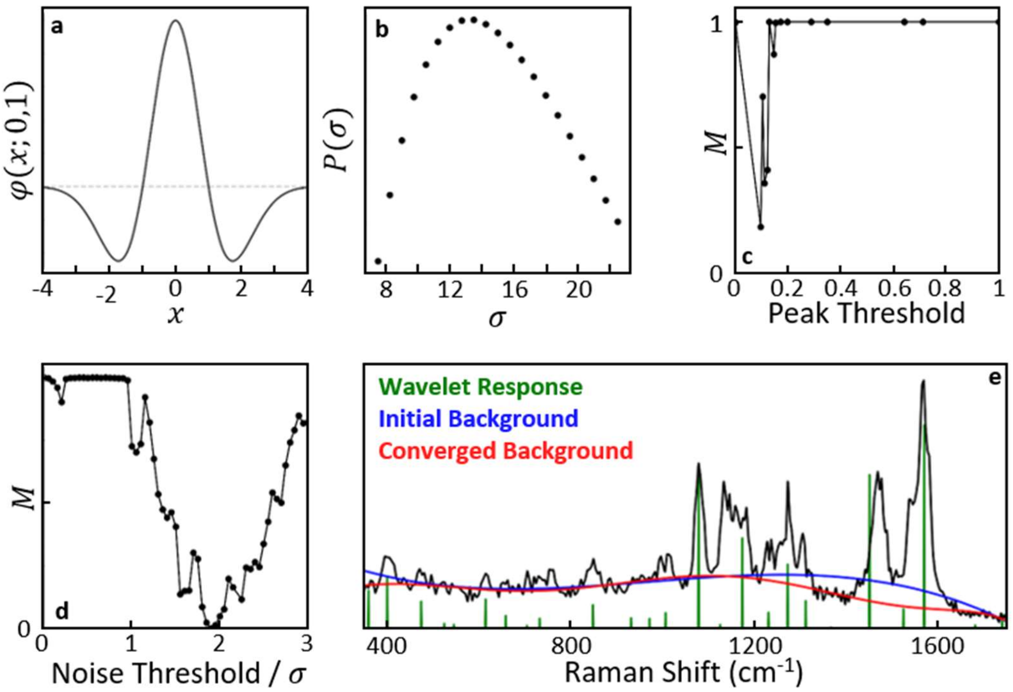

Figure S7 | Example background estimation. a, The Ricker wavelet, which can be applied to a spectrum to return a score **b,** of how well the spectrum is represented by peak-like structures of width σ. The wavelet can be used to estimate peak positions that can be masked out of the initial background estimate. **c,** Metric M for these backgrounds varies with the relative threshold wavelet response for a possible peak to be masked. The estimate with lowest M is taken. **d,** To iterate the background estimate, all points exceeding the current estimate by a noise threshold are masked out. This threshold is scanned to identify the estimate that minimises M. **e,** An example SERS spectrum, with possible peaks detected via wavelet analysis (green) defining an initial background estimate (blue), and iterated to generate the converged estimate background (red).

Spectral Scan Flattening

The persistent SERS from a scan of spectra, with backgrounds removed, can change slowly over time in intensity due mostly to drift in the experimental system. If these changes can be estimated and corrected, estimation of the persistent SERS over time becomes much simpler. This correction is defined by a map of values by which the spectrum scan is divided. This map does not have to be perfect, as its application is reversed once the persistent SERS has been estimated. Generating this map is described in detail here, but the high level process is also shown using the following flow diagram.

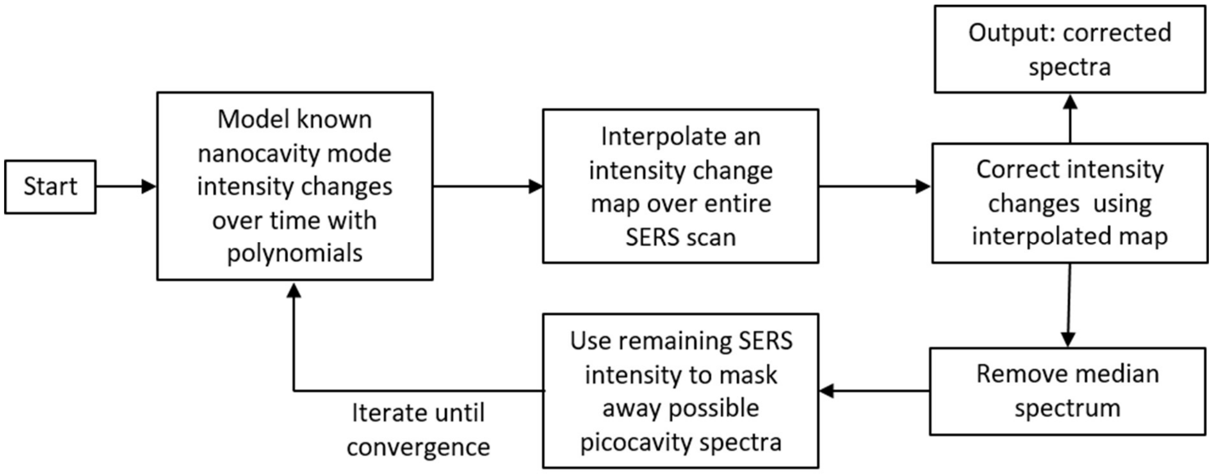

The map is defined by the intensities of known nanocavity peaks over time approximated with smooth polynomials and is interpolated between these known spectral positions using cubic splines. The SERS at nanocavity peak positions can be increased by a picocavity event, so such spectra must first be masked out with high sensitivity and ignored when defining the map. This mask is first initialised with no spectra labelled as possible picocavity spectra, and is updated in an iterative process. During this process, a correction map is generated by fitting the integrated SERS from nanocavity lines to polynomial curves of a given order while ignoring masked spectra. Different maps are generated depending on the polynomial order. To select an optimal order that does not over-fit, each scan of corrected and masked nanocavity spectra are compared to their median to define a loss for the correction quality. In general, this loss drops with increasing polynomial order due to the larger number of parameters. The point of highest curvature in the loss-order curve represents the threshold of diminishing returns for further increases to polynomial order and is selected. This correction map is used to correct the scan over spectra and the median nanocavity-labelled spectrum is removed. The total remaining SERS at each point in time is taken as a metric, formed from a time-dependant baseline that increases discretely during a picocavity event (Figure S8c). This baseline is removed using the background removal technique described above. A noise level for the corrected metric can be defined as the root mean square of all negative values. A new mask of possible picocavity spectra is defined where the metric surpasses a defined threshold (set here as twice the noise level). This can lead to a number of false single spectrum picocavity event identifications. As a single spectrum picocavity event is unlikely to have any significant effect on the resulting correction map, all single spectrum events are removed from the mask. This process is iterated until either the mask converges or a maximum iteration number is reached (here: 10 iterations).

With possible transient SERS masked away, the integrated SERS from each nanocavity line over time can be fit using polynomial functions of differing orders. The order for each line is set by minimising the Bayesian Information Criterion. This defines the final correction map (Figure S8).

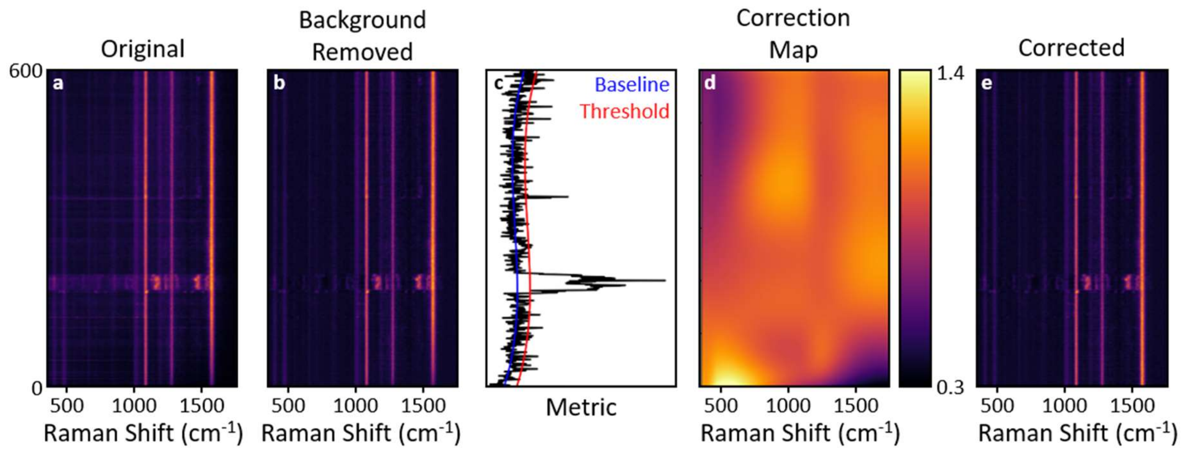

Figure S8 | Example background estimation. **a** An example scan of SERS spectra has **b,** the background estimated and removed. **c,** Metric used shows discrete changes in the scattering intensity on top of ignored slow changes in the nanocavity scattering. This is combined with a threshold to label spectra as possibly containing picocavity scattering. **d,** For the non-picocavity spectra, the total scattering from known nanocavity SERS lines is approximated as polynomial curves over time. These are spectrally interpolated using cubic splines to generate a correction map for the slow changes in nanocavity scattering. **e,** Dividing by this map flattens the nanocavity scattering response.

Estimating Nanocavity SERS

With the spectral scan corrected using the correction map, the persistent SERS should remain fairly static over time. However, some smaller variations can remain. To estimate the persistent nanocavity SERS, possible picocavity spectra must be removed from the scan with high sensitivity. The required sensitivity is higher here than during the definition of the correction map. Once these are removed, the remaining spectra are using to estimate the persistent SERS over all time using smoothing and linear interpolation. The processes of both masking out possible picocavity SERS and then generating the nanocavity SERS estimate are described in detail here, but the high level processes are also shown using the following flow diagrams.

Nanocavity Estimation from Masked Spectral Scan

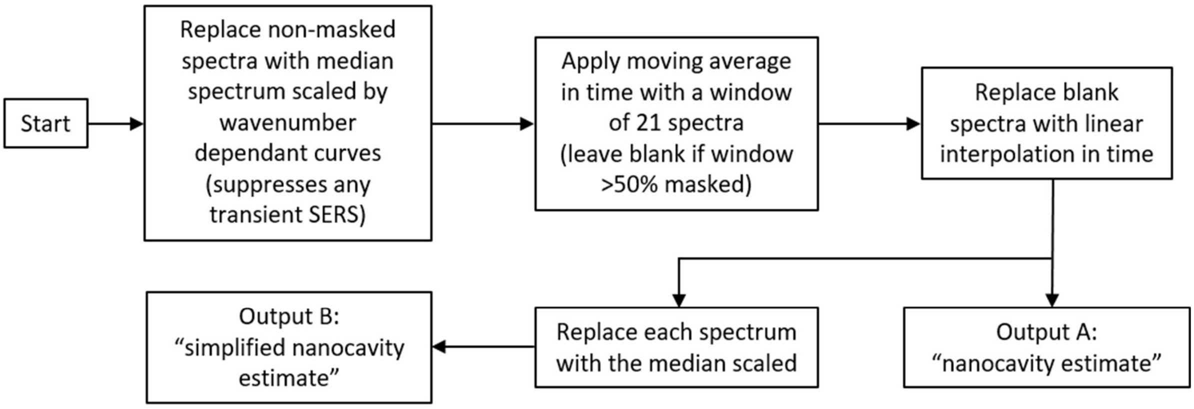

Defining Spectral Scan Mask

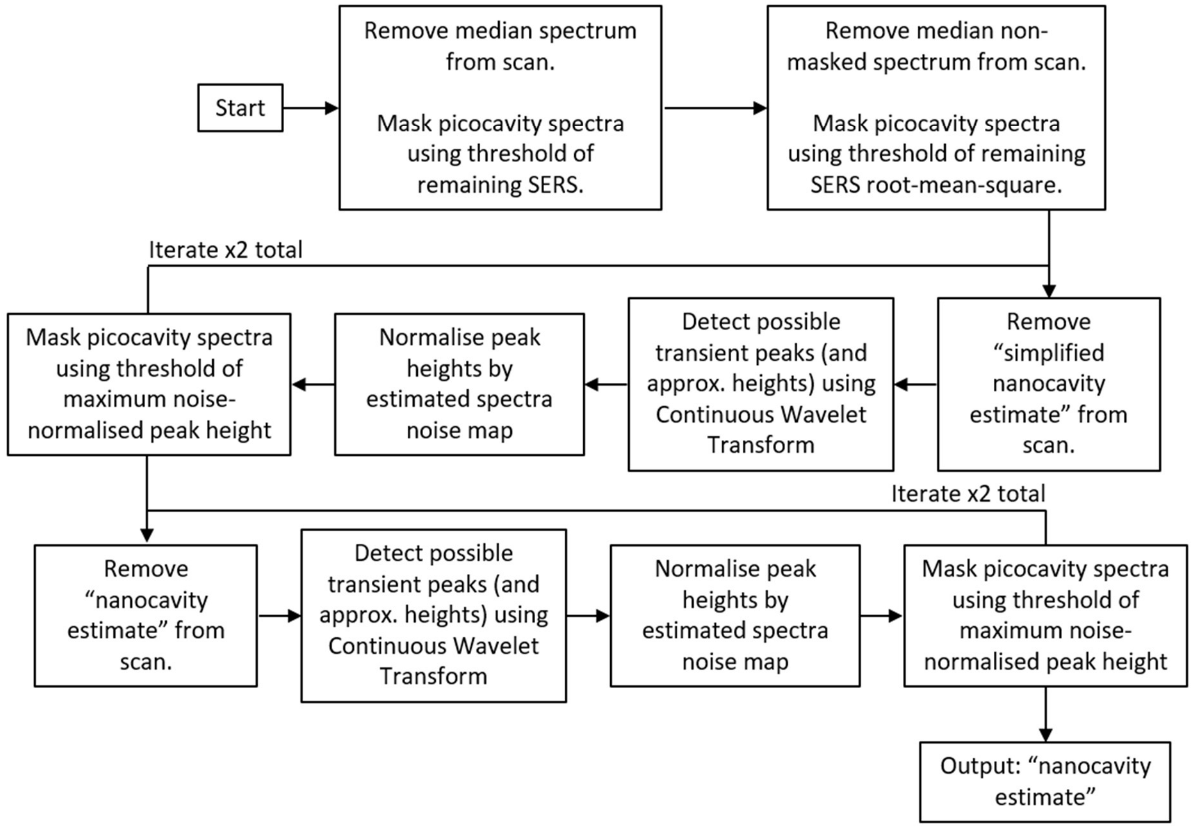

<u>Generating a nanocavity estimate from a picocavity mask:</u>
A given mask is generated for spectra that might represent picocavities. This must be converted into an estimate for the persistent SERS over all time. To achieve this, a moving average is applied to the non-masked spectra to smooth the persistent response before linear interpolation over time is used to fill in any gaps left after masking. However, if the mask misses a weak but long lived picocavity event, the moving average can incorporate this transient SERS into the nanocavity estimate that must be suppressed. This is artificially simulated in Figure S9a. This can be avoided by noting that each nanocavity spectrum should be very similar over time, and therefore well approximated by their median. Replacing each spectrum by this median multiplied by an intensity-dependent scaling curve (a cubic spline with anchor points at known nanocavity SERS peak positions), does not include weak transient peaks which are then successfully suppressed (Figure S9b).

With any transient peaks supressed, the moving average is applied. Here, a window size of 21 spectra is used. To prevent averaging an arbitrarily small number of spectra, if over 50% of the spectra within an averaging window are masked then the average is not carried out and that spectrum is left blank (Figure S9c). All of these blank spectra are then reconstructed using linear interpolation in time (Figure S9d).

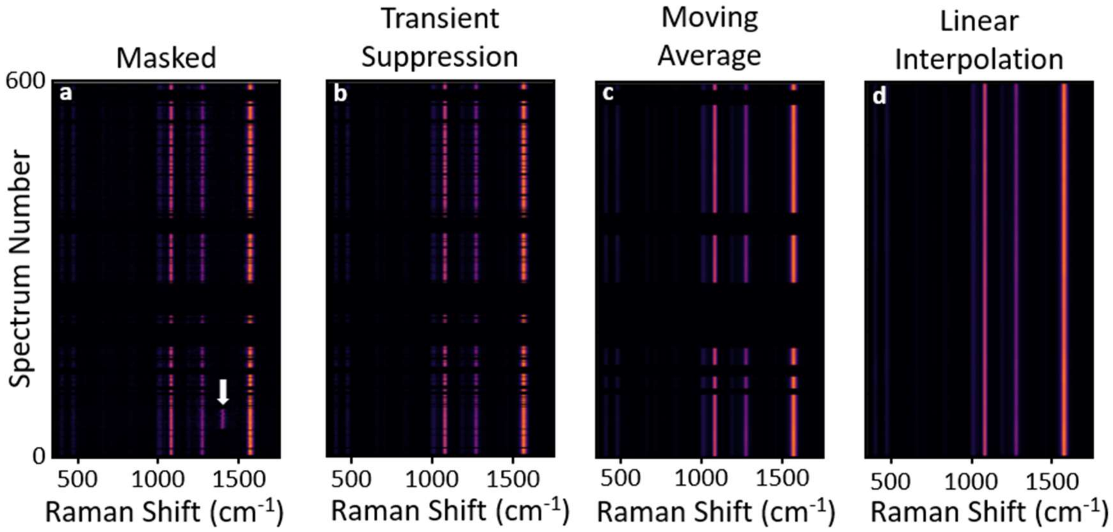

Figure S9 | Example nanocavity estimation. a Example flattened scan of SERS spectra with certain spectra masked out as possibly representing picocavities. A synthetic weak transient peak (white arrow) is added to demonstrate how these are handled. **b,** Each unmasked spectrum is replace by their median multiplied by a spectrally dependant curve, recreating the nanocavity peaks while supressing small transient SERS events. **c,** A moving average is applied in time with a window size of 21 spectra. If this window is less than 50% filled with unmasked spectra than the spectrum is left blank, leaving gaps in the resulting scan. **d,** These gaps are filled using linear interpolation in time.

Note that if the mask is in an initial and crude state that does not remove transient SERS spectra well, this nanocavity estimate may contain some undesirable features. To handle this, a “simplified nanocavity estimate” is defined where each nanocavity estimate spectrum is replaced with a scaled version of the median spectrum.

<u>Initialising the picocavity mask:</u>

The mask of possible picocavity spectra is initialised using a simple threshold of the total scattering. The median spectrum is removed from the scan and what remains at each point in time is summed to generate a metric. In the example shown in Figure S10a-b, this median is already a good estimate for the nanocavity SERS but this is not true in general. This metric is modelled as drawn from a Gaussian distribution with positive outliers due to picocavity events. For an initial high sensitivity mask, all spectra with a metric above the centre of this Gaussian are labelled as picocavity Figure S10c).

A median of the remaining non-masked spectra is taken and removed from the scan instead (Figure S10d). The probability that picocavities could meaningfully influence this median spectrum is much lower and the metric is altered to the root mean square (RMS) of the remaining SERS at each point in time. A mask threshold is set at twice the standard deviation above the centre of the Gaussian that the metric baseline represents (Figure S10e). This process can be iterated, but here is only applied

once more to define the initial mask of possible picocavity spectra. This mask initialisation is crude but computationally cheap.

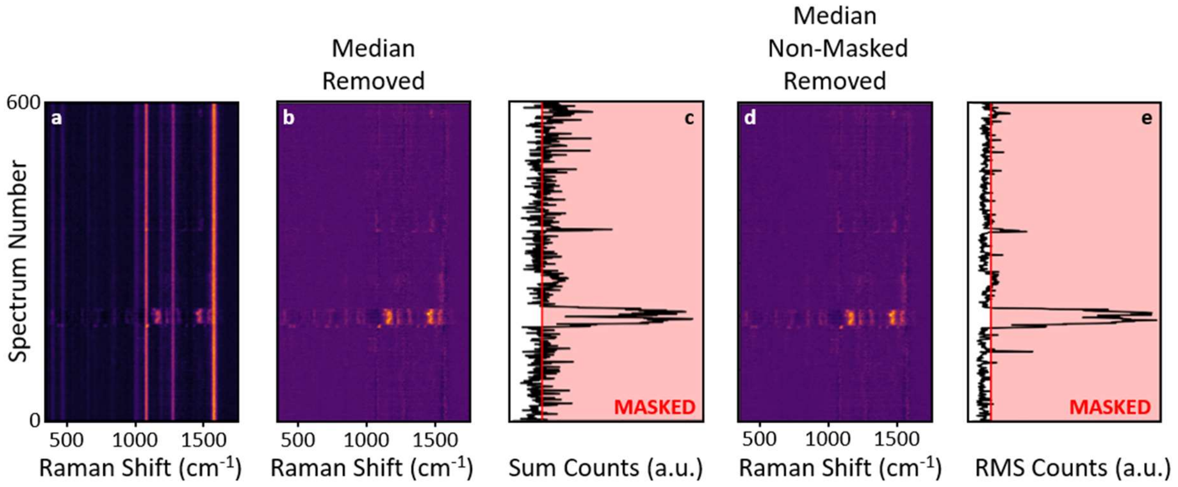

Figure S10 | Mask Initialisation. a Example flattened scan of SERS spectra has **b,** the median spectrum removed. **c,** The remaining summed SERS is used as a picocavity metric. Any spectrum above the baseline of this metric is masked as a possible picocavity. **d,** The median of the unmasked spectra is removed from the SERS scan and **e,** a new metric is formed from the root mean square (RMS) of the remaining SERS. Any spectrum exceeding one noise level above the baseline is masked as a possible picocavity.

Iterating the picocavity mask:

To iterate and improve the mask of possible picocavity spectra, the current nanocavity estimate is removed from the scan (Figure S11a). This is either the full or "simplified" estimate (defined above) depending on the number of iterations undertaken so far (discussed below). Using a simple intensity threshold for possible picocavity detection risks missing weak events. Instead, we here use a Continuous Wavelet Transform (CWT) to attempt to pick out peaks in the remaining SERS. This uses the Ricker wavelet Figure S7a) and is applied using the characteristic width of SERS peaks in the nanocavity estimate. This extracts the positions and intensities of possible picocavity peaks.

With estimates for the background, nanocavity and picocavity response of the scan of SERS spectra and prior knowledge of the instrument response function correction curves and the correction map that was applied, a noise map for the spectrum scan can be defined (Figure S11b). This map assumes that both the photon counts at the detector and the spectrally uniform dark counts measured follow Poisson distributions. The greater noise at the spectral positions of removed nanocavity peaks makes it more likely here to incorrectly detect possible picocavity peaks in the noise. Dividing the heights of all possible detected picocavity peaks by the noise map corrected for this. A picocavity metric is defined as the largest value of this height : noise ratio at each point in time. Compared to a sum of SERS scattering, this metric is sensitive to weaker picocavity events (Figure S11c). Again, this metric is modelled as a Gaussian distribution with positive outliers and picocavity events are defined using a threshold one standard deviation above the distribution centre. All single spectrum picocavity events are relabelled as nanocavity spectra.

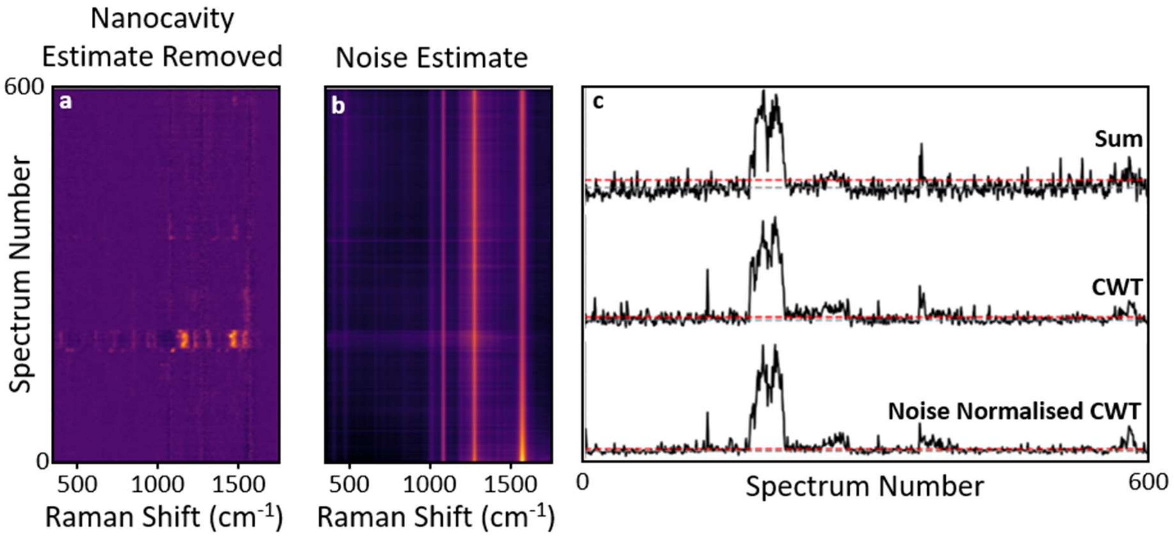

Figure S11 | Picocavity Mask Iteration. a, The current nanocavity SERS estimate is removed from the spectrum. **b,** An estimate for the scan noise profile in absence of the transient SERS. **c,** A comparison of metrics for defining possible picocavities. These include the sum of remaining SERS, the maximum response after a Continuous Wavelet Transform, and this same response normalised by the noise profile. The Normalised CWT is used here, as weak events are less dominated by metric noise. Grey lines indicated metric baselines and red lines indicate one noise level above the baseline.

A simple and computationally cheap method to check and correct glaring errors in the picocavity mask is to compare the spectra during a picocavity detection with the nanocavity spectra before and after. If these spectra are more similar to the average nanocavity spectrum either side of the detection than their own average, this picocavity detection is clearly in error and the mask is updated. This will only catch a certain fraction of nanocavity spectra labelled incorrectly as picocavity events, but is very cheap to compute and always improves the mask.

Once the picocavity mask in initialised, it is iterated using the “simplified nanocavity estimate” twice. It is iterated twice more using the full nanocavity estimate. Re-multiplying the final result by the correction map (Figure S12c) provides the final estimate for the persistent nanocavity SERS.

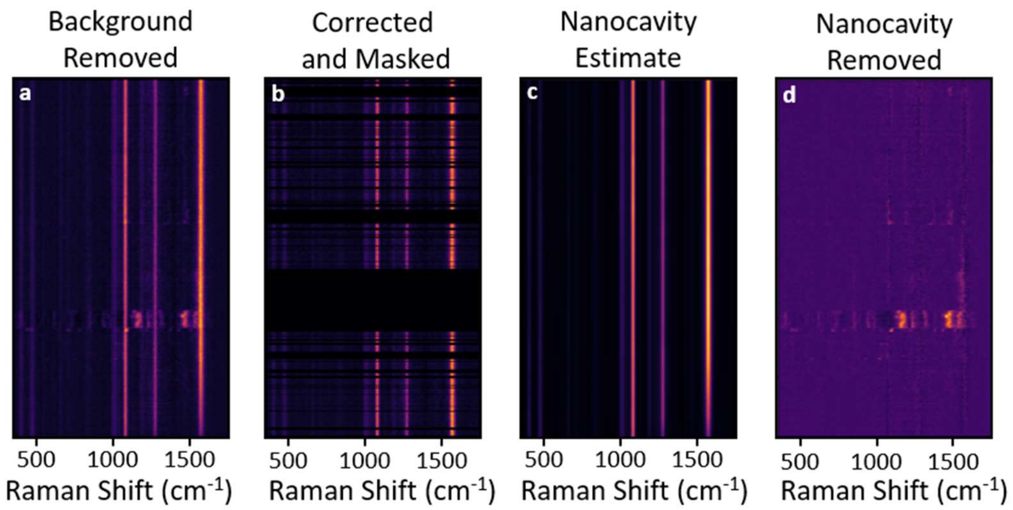

Figure S12 | Example nanocavity estimation. a, Example time scan of SERS spectra with background estimate removed. **b,** This is divided by a correction map and possible picocavity-containing spectra are masked out. **c,** A moving average is used to estimate the nanocavity over time, with gaps filled in using linear interpolation. The correction map application is reversed to give the final nanocavity SERS estimate. **d,** This is then removed from the scan of spectra to isolate the picocavities.

Defining Picocavity Events

With background and nanocavity scattering estimates removed from a scan of spectra, what remains is 'transient scattering'. Sets of consecutive spectra must be defined as representing picocavity events. A robust way to achieve this is attempting to extract the picocavity peaks from the spectra. This is described in detail here, but the high level process is also shown using the following flow diagram.

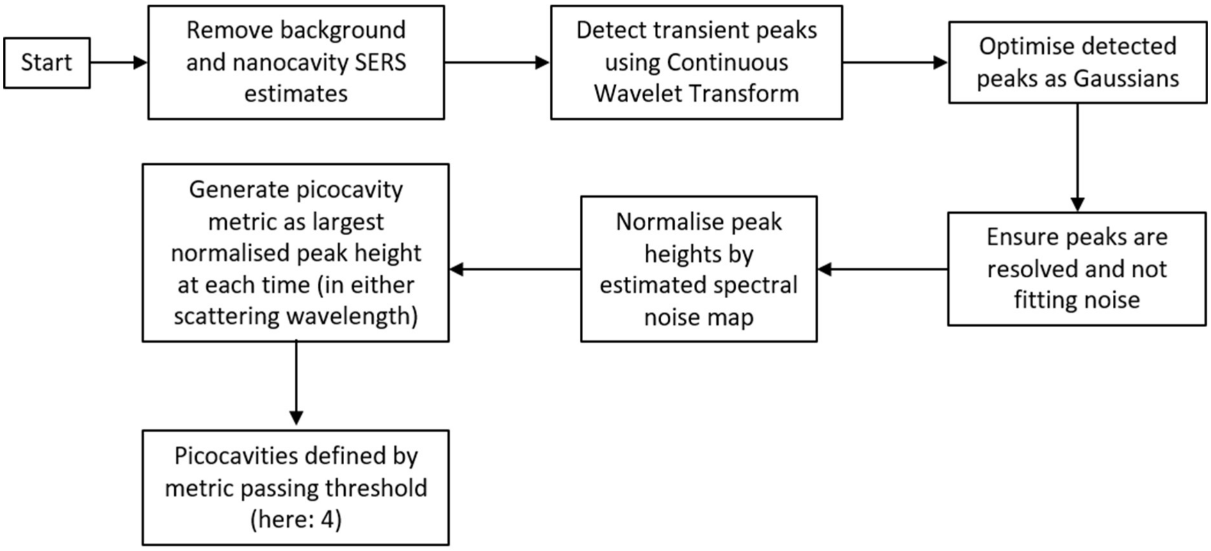

To reduce computational complexity, possible peak positions are limited to discrete Raman-shifted spectral positions. Peaks are initially estimated using wavelet analysis and modelled as Gaussian peaks to reconstruct the scan (Figure S13). A second wavelet analysis on the scan with these optimised peaks removed can uncover any peaks that may have been initially missed. Simple rules are implemented to guide peak optimisation. Optimised peaks must be resolvable, defined by peaks being separated by a larger spectral distance than the sum of their width parameters. The region being described by a given

peak must resemble a peak more than it resembles a polynomial of order ≤ 2 as defined by the Bayesian Information Criterion. This prevents small remaining scattering variations in the background being described as weak broad peaks. With a given set of optimised picocavity peaks, a noise map for the scan can be defined. This is important as the noise increases at the spectral positions of excised large nanocavity peaks, generating 'phantom' apparently-narrow peaks in the nanocavity-removed spectra. To mitigate these, any peak with a height below the noise level at its spectral location can be safely discarded. By dividing the spectra (with background, nanocavity and optimised picocavity peaks removed) by the noise map, what remains should be a map of normalised noise. Using wavelet analysis, the characteristic spectral scale of the noise can be extracted. Any optimised peaks with a width comparable to this can be removed as noise. This cycle of the removing peaks that do not fit these criteria and re-optimising those that remain can be iterated.

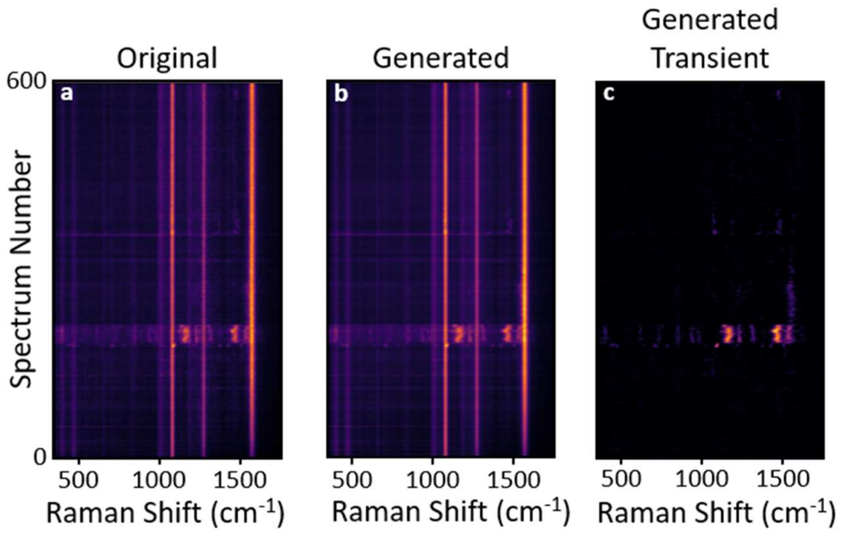

Figure S13 | Transient Peak Extraction. a, Example time series of SERS spectra. **b,** Reconstruction of this series as a sum of SERS background, persistent nanocavity SERS and transient SERS peaks modelled with Gaussian functions. **c,** These modelled transient peaks now isolated from the rest of the SERS.

A spectrum is defined as part of a picocavity event if it contains a detected peak with height (normalised by the noise map) meeting a given threshold. In Figure S14, this threshold is set to 4. In analysing the experimental results, we are comparing SERS scattering taken at 633 nm and 785 nm simultaneously. A point in time is taken to be part of a picocavity event if the threshold is met in either wavelength. A set of consecutive points in time that all trigger the threshold defines a picocavity event.

To compare scattering in both wavelengths, the picocavity scattering must first be normalised to the nanocavity scattering. The nanocavity scattering in the two wavelengths varies not only due to the difference in laser power coupled into the cavity, but also the spectral dependence of the NPoM out-coupling. In addition, chromatic aberration means that the spectral widths of the 633 nm SERS lines are broadened with respect to those at 785 nm. To correct for this, the 785 nm scattering is artificially broadened using a Gaussian kernel. The resulting broadened spectra can then be scaled in intensity by a cubic spline with control points at the position of known nanocavity lines. This broadening and cubic spline are optimised to match the estimated nanocavity scattering at 785 nm to that estimated at 633 nm. This same broadening and scaling can then be applied to the transient spectra, normalising for both in-coupled laser power and differences in out-coupling efficiencies at both wavelengths (Figure S14).

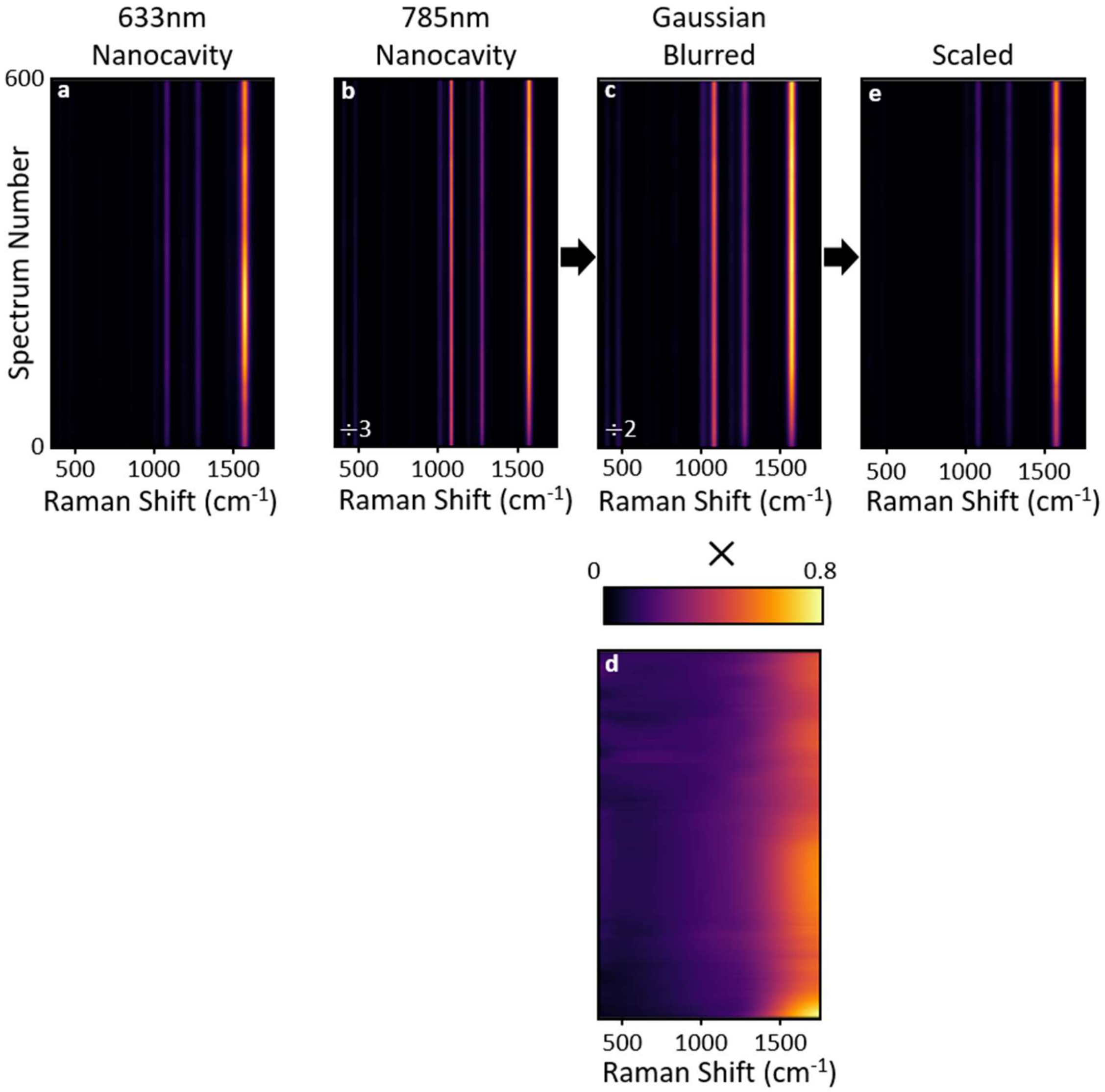

Figure S14 | Matching nanocavity SERS. a, 633nm nanocavity SERS and **b,** 785nm nanocavity SERS differ due to chromatic aberration and difference in incident laser powers and in-coupling into the plasmonic nanocavity. These are matched by applying a **c,** Gaussian Blur and **d,e,** spectrally and time dependant scaling. This corrects for these differences.

If the background and nanocavity-subtracted emission from a spectrum is very small or non-existent, noise can render the summed transient scattering negative. In these cases, the scattering is set to zero. The total scattering during a picocavity event can now be extracted for both 633 nm (S_{633}) and 785 nm (S_{785}). For each event, the final emission ratio metrics can be defined as

$$R \equiv \frac{S_{785}}{S_{633}}, \quad \rho \equiv \frac{S_{785} - S_{633}}{S_{785} + S_{633}}. \tag{10}$$

For events that are weak in absolute scattering intensity, the uncertainty due to noise is likely to dominate these metrics. For this reason, the results from these low intensity events are likely to obscure the underlying distribution of ρ. Therefore, a threshold of detected picocavity event intensity must be implemented. For all spectra within the dataset, the ratio of transient to total scattering can be calculated. For each point in time, the largest ratio from each wavelength is taken. Separate

distributions can be constructed for spectra labelled as picocavity or not (Figure S15a). While it is initially counter-intuitive that the distribution for non-picocavity spectra is not maximised at zero, this is a direct result of taking the maximum ratio from each wavelength. These distributions overlap, and can be combined to generate a curve of event intensity versus confidence that the event can be resolved from noise (Figure S15b). As this confidence is increased, more picocavity events are discarded. Without discarding any events, the distribution of ρ is rather flat as the high uncertainty associated with weak events masks the true distribution. As the confidence threshold is raised, a resolvable distribution shape for ρ emerges (Figure S15c-f). In this work, a confidence threshold of 90% is used.

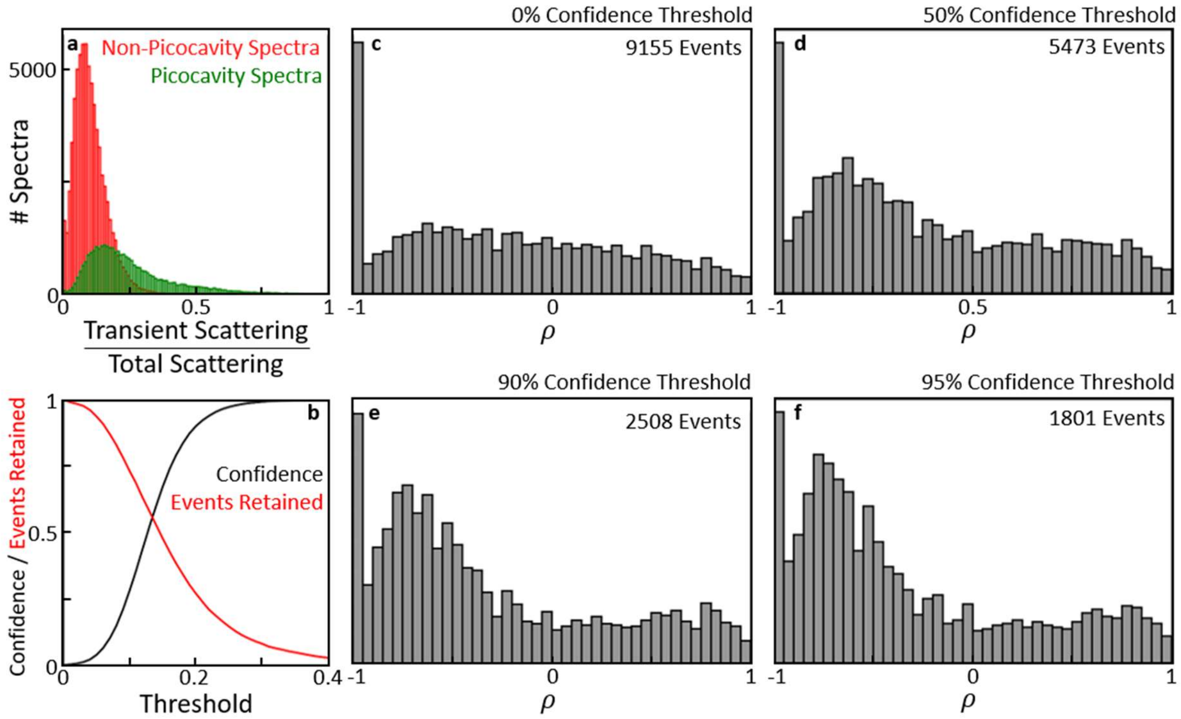

Figure S15 | Setting the picocavity intensity threshold. a, For the entire dataset of SERS spectra, the distribution of transient scattering / total scattering for events labelled as containing and not-containing a picocavity overlap. **b,** This is used to define a curve for the confidence that a picocavity event is resolvable from the noise. Discarding events based on the threshold generates difference histograms for ρ based on a minimum of **c**, 0% **d,** 50% **e,** 90% and **f,** 95% confidence.

Analysing the data in this way, the height threshold for defining a spectrum as part of a picocavity event can be varied, and distributions for picocavity formation rate and lifetime can then be constructed (Figure S16). If this threshold is small, the total number of events detected is low as picocavity events are all blurred together. As the threshold is raised, the total number of events increases to a maximum before decreasing again when true picocavity events start to be discarded. The time between picocavity events follows an exponential distribution as picocavity events are mutually independent. As the threshold is raised, this becomes a double exponential as picocavity events become split into multiple events artificially abutting each other. A peak threshold of 4 is used in the manuscript, optimising the picocavity identification before the onset of this double exponential behaviour.

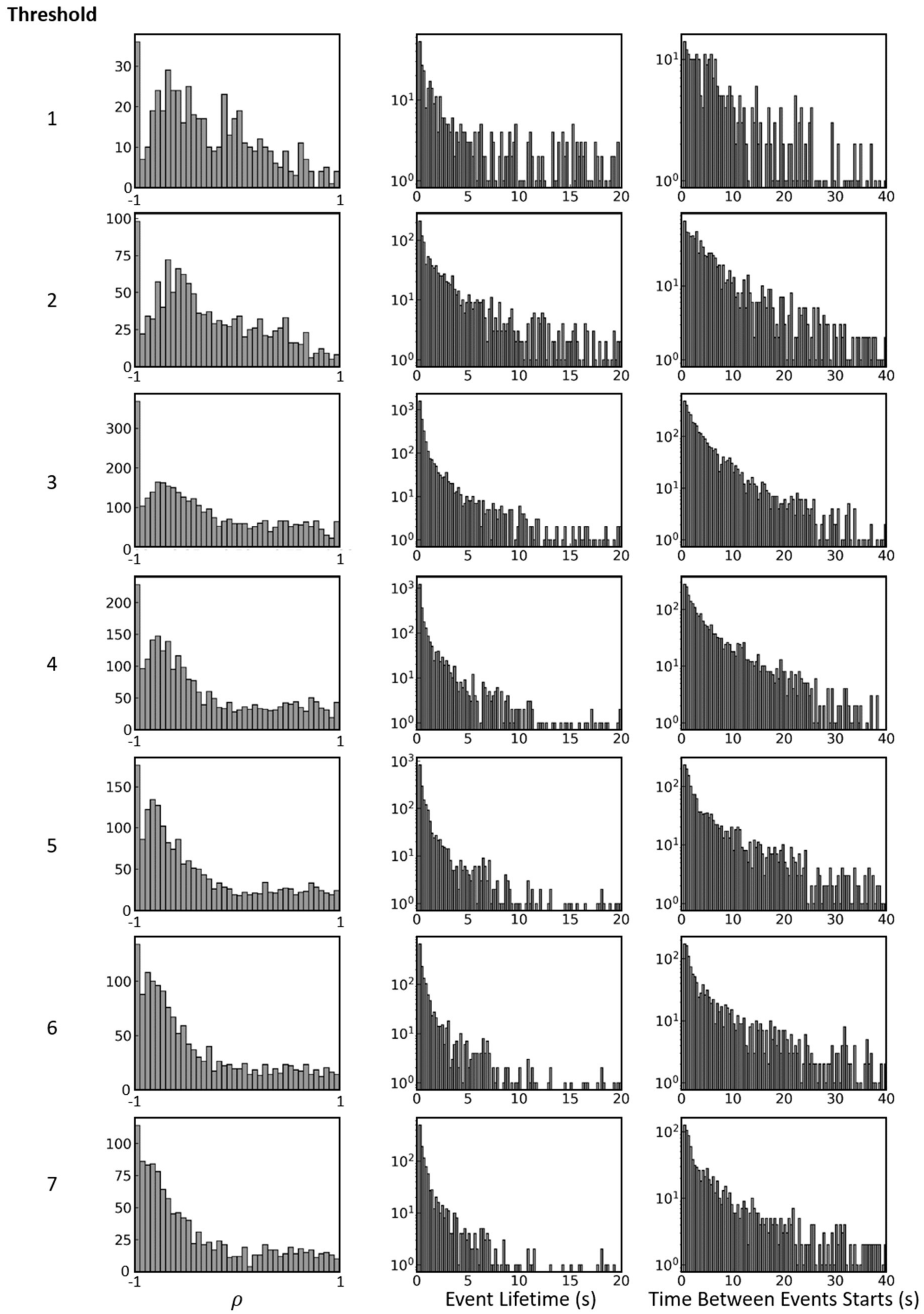

Figure S16 | Distributions with different picocavity detection thresholds. With varying scattering thresholds 1-7 for identifying picocavity spectra, the total number of events defined changes. This alters the histograms of ρ and the distributions of picocavity event lifetimes and picocavity formation rates.

Model Derivation

SERS scattering from the NPoM system is described here in terms of 2D surface S representing the lateral area under the nanoparticle facet. Adatoms can form at any position on S without distinction of whether they form on the facet or mirror. At position $\underline{x}$ on S, the local field strength at wavelength λ is defined as $E(\underline{x},\lambda) = A_\lambda\,\psi(\underline{x},\lambda)$ where $\psi(\underline{x},\lambda)$ is dimensionless and describes the spatial field profile. The SERS from this location is $kE^4(\underline{x},\lambda)$ where k is a constant. A picocavity provides (for the purposes of SERS) an effective enhancement α_λ to the local field strength. Therefore, the SERS from a picocavity event, normalised by the total SERS from the entire NPoM nanocavity, is given by

$$\text{Picocavity SERS} \propto \frac{k\alpha_\lambda^4 E^4(\underline{x},\lambda)}{k\int_S E^4(\underline{x},\lambda)d^2\underline{x}} = \frac{\alpha_\lambda^4 \psi^4(\underline{x},\lambda)}{\int_S \psi^4(\underline{x},\lambda)d^2\underline{x}}. \tag{11}$$

The ratio of normalised SERS at wavelength λ_1 compared to λ_2 is therefore given by

$$R(\underline{x}) = \frac{\alpha_{\lambda_1}^4 \psi^4(\underline{x},\lambda_1)}{\int_S \psi^4(\underline{x},\lambda_1)d^2\underline{x}} \div \frac{\alpha_{\lambda_2}^4 \psi^4(\underline{x},\lambda_2)}{\int_S \psi^4(\underline{x},\lambda_2)d^2\underline{x}}$$

$$= \frac{\mathrm{N}_4}{\alpha^4}\frac{\psi^4(\underline{x},\lambda_1)}{\psi^4(\underline{x},\lambda_2)} \text{ where } \alpha \equiv \frac{\alpha_{\lambda_2}}{\alpha_{\lambda_1}} \text{ and } \mathrm{N}_n \equiv \frac{\int_S \psi^n(\underline{x},\lambda_2)\,d^2\underline{x}}{\int_S \psi^n(\underline{x},\lambda_1)\,d^2\underline{x}}. \tag{12}$$

This expression is conveniently expressed in terms of ratios of parameters for each wavelength. More specifically, these are the ratio of normalised local nanocavity field intensities and the ratio of picocavity field enhancements.

Experimentally, weak picocavity SERS is likely to be suppressed by noise and perhaps partially discarded as part of the nanocavity SERS background. To account for this in the model, picocavity SERS must be modified by a suppressing function $s(I) \leq I$ such that

$$\begin{gathered} s(I \to 0) = 0 \\ s(I \to \infty) = I \\ s(I)\,/I \text{ is monotonic} \end{gathered} \tag{13}$$

leading to the expression

$$R(\underline{x}) = \left(\frac{A_{\lambda_2}}{A_{\lambda_1}}\right)^4 \frac{\int_S \psi^4(\underline{x},\lambda_2)d^2\underline{x}}{\int_S \psi^4(\underline{x},\lambda_1)d^2\underline{x}} \frac{s\left(k\alpha_{\lambda_1}^4 A_{\lambda_1}^4 \psi^4(\underline{x},\lambda_1)\right)}{s\left(k\alpha_{\lambda_2}^4 A_{\lambda_2}^4 \psi^4(\underline{x},\lambda_2)\right)}$$

$$= \left(\frac{A_{\lambda_2}}{A_{\lambda_1}}\right)^4 \mathrm{N}_4 \frac{s\left(k\alpha_{\lambda_1}^4 A_{\lambda_1}^4 \psi^4(\underline{x},\lambda_1)\right)}{s\left(k\alpha_{\lambda_2}^4 A_{\lambda_2}^4 \psi^4(\underline{x},\lambda_2)\right)}. \tag{14}$$

By noting that the ratio of total optical power coupled into the NPoM cavity at λ_2 compared to λ_1 is given by $L \equiv \left(\frac{A_{\lambda_2}}{A_{\lambda_1}}\right)^2 \mathrm{N}_2$, this can be re-expressed as

$$R(\underline{x}) = \frac{\mathrm{N}_4}{{\mathrm{N}_2}^2} L^2 \frac{s\left(k\alpha_{\lambda_1}^4 A_{\lambda_1}^4 \psi^4(\underline{x},\lambda_1)\right)}{s\left(k\alpha_{\lambda_2}^4 A_{\lambda_2}^4 \psi^4(\underline{x},\lambda_2)\right)}. \tag{15}$$

Function s can take many ad hoc forms, but a reasonable goal here is to minimise the total number of unknown parameters. This can be achieved using the overtly simple definition $s(I\ ;\ I_0) \equiv \max(I - I_0\ , 0)$. Below a threshold, this definition sets the detected SERS light to zero as it is completely dominated by experimental noise. Above this threshold, the relative suppression decreases with input magnitude. Using this definition,

$$R(\underline{x}) = \frac{\mathrm{N}_4}{{\mathrm{N}_2}^2} L^2 \; \frac{\max\left(k\alpha_{\lambda_1}^4 A_{\lambda_1}^4 \psi^4(\underline{x}, \lambda_1) - I_0, 0\right)}{\max\left(k\alpha_{\lambda_2}^4 A_{\lambda_2}^4 \psi^4(\underline{x}, \lambda_2) - I_0, 0\right)}$$

$$= \frac{\mathrm{N}_4}{{\mathrm{N}_2}^2} L^2 \; \frac{\max\left(\psi^4(\underline{x}, \lambda_1) - \delta, 0\right)}{\max\left(\alpha^4 \left(\frac{L}{\mathrm{N}_2}\right)^2 \psi^4(\underline{x}, \lambda_2) - \delta, 0\right)}, \quad \delta \equiv \frac{I_0}{k\alpha_{\lambda_1}^4 A_{\lambda_1}^4} \tag{16}$$

with the effective noise threshold now controlled through parameter δ and L modifying the relative impact of noise on each wavelength. As required, this suitably returns to the noiseless expression in the limit $\delta \to 0$. This expression is not necessarily defined at all $\underline{x}$, since at some positions SERS is too weak (in either wavelength) to be detected experimentally.

Optimising Picocavity Models

The experimental histogram for ρ can be numerically matched by optimising the model described in "Defining Picocavity Events" for varying probability density functions (PDFs) describing the formation probability of picocavities at different locations. The field profiles at each scattering wavelength are taken from Finite Difference Time Domain (FDTD) simulations of the nanoparticle-on-mirror (NPoM) structure formed from an 80 nm diameter AuNP truncated to give a $R = 10$ nm radius circular facet. Due to symmetry, only the field dependence in the radial distance r from the facet centre impacts the model here with the angular dependence separable and wavelength independent. These distributions differ depending on whether the structure is excited at a normal or high incidence angle. Assuming uniform spatial probability for picocavity formation, the high angle distributions are unable to approximate the experimental results and the normal-excited field distribution (as produced by focussing in the pump laser through the objective lens) is used here instead (Figure S17).

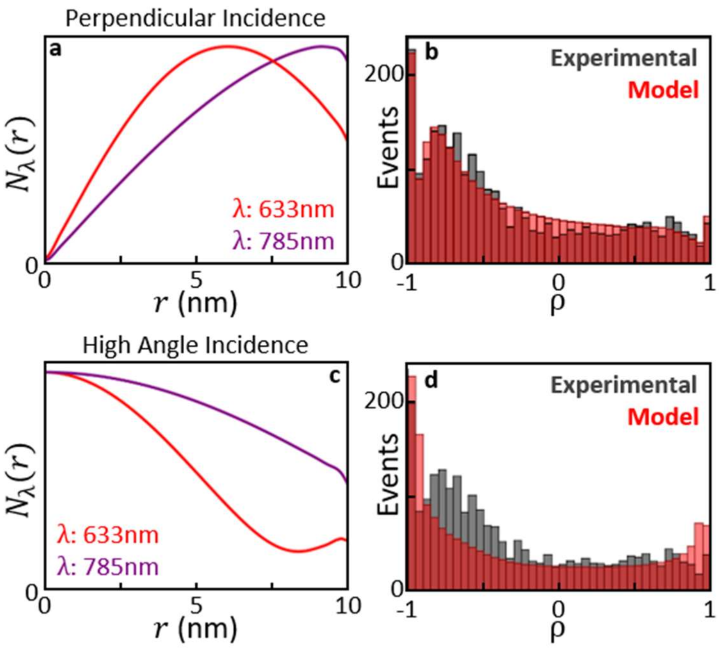

Figure S17 | Comparing Field Distributions. a, FDTD field distributions within the NPoM cavity for both SERS wavelengths vs distance (r) from the AuNP facet centre under normally-incident laser irradiation. **b,** Assuming uniform adatom generation rates at all points on the facet, these distributions can be used to recreate the shape of the experimental histogram for ρ. **c,** Field distributions for high-angle illumination, **d**, which are unable to recreate the experimental results.

Various PDFs for the spatial distribution of picocavity generation are optimised, with these PDFs denoted as $P(r)dr \equiv r\, f(r)\, dr$ which pre-defines the geometric factor $\propto r$. The energy barrier for picocavity formation is denoted as $B(r)$. This is considered to either be constant or described by a sigmoid

$$B(r) = K_1 \left(1 + e^{-\frac{r-R}{C}}\right)^{-1} + K_2 \tag{17}$$

where K_1, K_2, C are constants and $B(r) \geq 0$. This allows for an energy barrier drop near the facet edge where the average surface atom coordination number is lower. In the Thermal Model, $f(r) \propto e^{-B(r)/kT}$. In the Optical Model, this energy barrier is suppressed by the local optical intensity $I(r)$ such that $f(r) \propto \exp\left\{-\frac{B(r)/kT}{I(r)}\right\}$ at higher powers. The total incoherently-summed intensity $I(r)$ is a linear combination of the intensities at each wavelength such that

$$I(r) \propto W\, \frac{\psi^2(r,\lambda_1)}{\int \psi^2(r,\lambda_1)\, r\, dr} + (1-W)\frac{\psi^2(r,\lambda_2)}{\int \psi^2(r,\lambda_2)\, r\, dr}, \quad 0 \leq W \leq 1 \tag{18}$$

which is normalised to unity at maximum. Note that for the Optical Model, this means that $B(r)$ is expressed relative to the maximum intensity coupled into the NPoM cavity. The final parameter varied is the weighting W, which depends on the λ_2:λ_1 ratios of optical power coupled into the NPoM cavity (L) and on the effective picocavity field enhancement (α). Two forms are considered here: $W = (1+L)^{-1}$ and $W = (1+\alpha^2 L)^{-1}$. The latter form includes the local picocavity near-field enhancement in the picocavity generation.

Each optimised model is shown below, for the specific form of $B(r)$ and W. The direct comparison between the experimental and modelled histograms of ρ can be re-expressed by inverting the

experimental values of ρ into adatom radial positions within the model and comparing directly to the input $f(r)$.

i) Thermal Model. $B(r)$ = constant

Optimised Parameters: α=0.81, L=2.00, δ=0.43

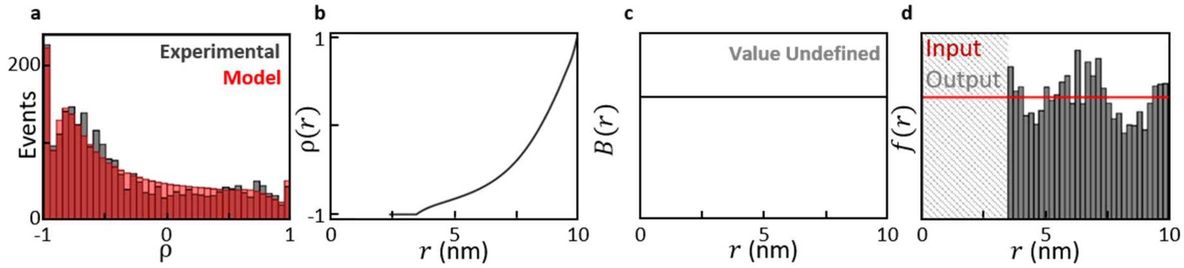

Figure S18 | Optimised Model. a, Histogram of modelled ρ optimised to experimental distribution. **b,** Optimised ρ with radial distance from facet centre r. Undefined where not present. **c,** Model $B(r)$. **d**, Model $B(r)$ (Input) against inverted distribution from optimised model (Output). Inversion undefined where shaded.

ii) Thermal Model. $B(r)$ = sigmoidal

Optimised Parameters: α=0.86, L=1.99, δ=0.43, $B(R) - B(0)$=0.97k_BT, C=61.5 nm

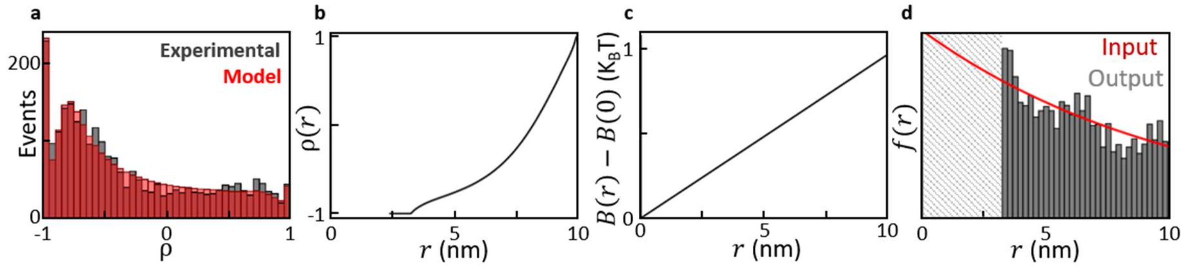

Figure S19 | Optimised Model. a, Histogram of modelled ρ optimised to experimental distribution. **b,** Optimised ρ with radial distance from facet centre r. Undefined where not present. **c,** Model $B(r)$. **d**, Model $B(r)$ (Input) against inverted distribution from optimised model (Output). Inversion undefined where shaded.

iii) Optical Model. $B(r)$ = constant, $W = (1 + L)^{-1}$

Optimises to $B(r)$ = 0, becoming (i).

iv) Optical Model. $B(r)$ = sigmoidal, $W = (1 + L)^{-1}$

Optimised Parameters: α=0.85, L=0.74, δ=0.41, $B(0)$=12.5, $B(R)$=8.23, C=0.51 nm

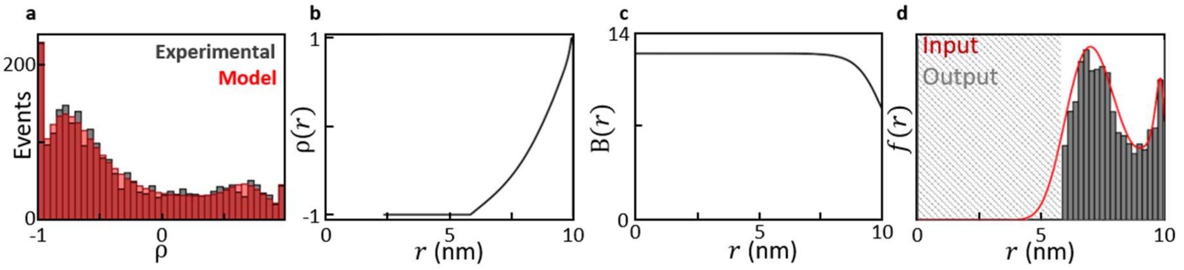

Figure S20 | Optimised Model. a, Histogram of modelled ρ optimised to experimental distribution. **b,** Optimised ρ with radial distance from facet centre r. Undefined where not present. **c,** Model $B(r)$. **d**, Model $B(r)$ (Input) against inverted distribution from optimised model (Output). Inversion undefined where shaded.

v) Optical Model. $B(r)$ = constant, $W = \left(1 + \alpha^2 L\right)^{-1}$

Optimises to $B(r)$ = 0, becoming (i).

vi) Optical Model. $B(r)$ = sigmoidal, $W = \left(1 + \alpha^2 L\right)^{-1}$

Optimised Parameters: α=0.86, L=0.76, δ=0.42, $B(0)$=11.6 , $B(R)$=7.12, C=0.62 nm

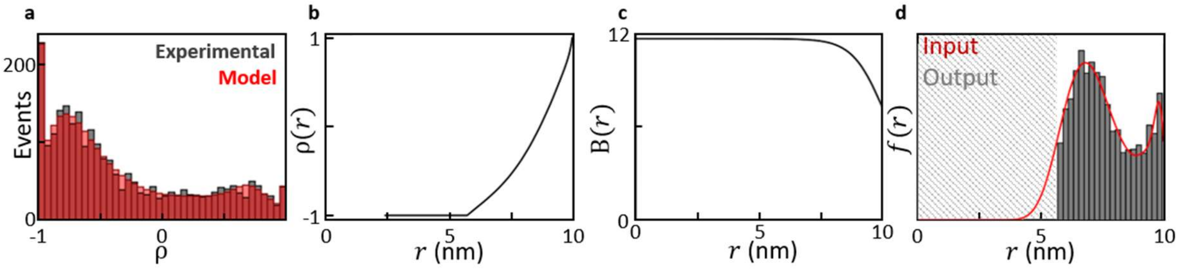

Figure S21 | Optimised Model. a, Histogram of modelled ρ optimised to experimental distribution. **b,** Optimised ρ with radial distance from facet centre r. Undefined where not present. **c,** Model $B(r)$. **d**, Model $B(r)$ (Input) against inverted distribution from optimised model (Output). Inversion undefined where shaded.

These models can be re-optimised while fixing L (see “Fixing L”), which lowers the number of free parameters.

vii) Thermal Model. $B(r)$ = constant, fixed L.

Optimised Parameters: α=0.82, L=1.16, δ=0.12

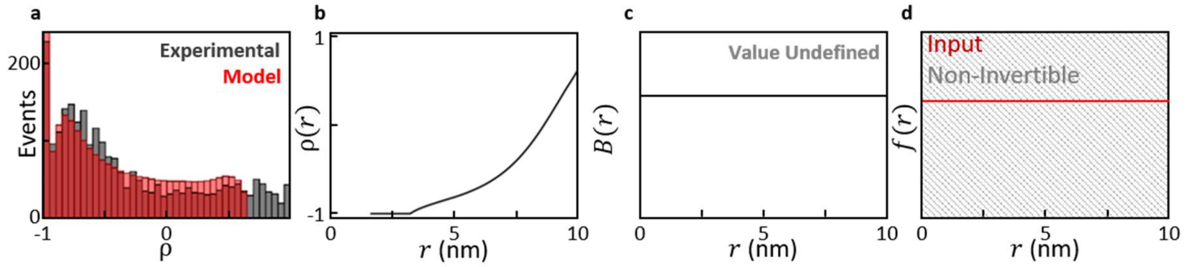

Figure S22 | Optimised Model. a, Histogram of modelled ρ optimised to experimental distribution. **b,** Optimised ρ with radial distance from facet centre r. Undefined where not present. **c,** Model $B(r)$. **d**, Model $B(r)$ (Input). Inverted distribution from the model is undefined.

viii) Thermal Model. $B(r)$ = sigmoidal, fixed L.

Optimised Parameters: α=0.90, L=1.16, δ=0.11, $B(R)-B(0)$=1.10k_BT , C=2.04 nm

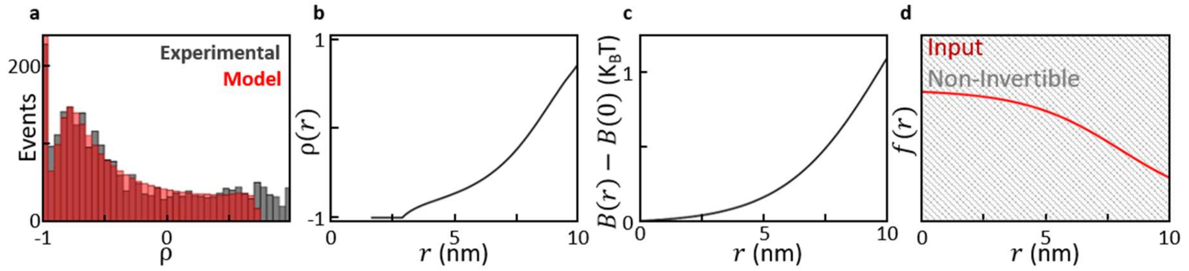

Figure S23 | Optimised Model. a, Histogram of modelled ρ optimised to experimental distribution. **b,** Optimised ρ with radial distance from facet centre r. Undefined where not present. **c,** Model $B(r)$. **d**, Model $B(r)$ (Input). Inverted distribution from the model is undefined.

ix) Optical Model. $B(r)$ = constant, $W=(1+L)^{-1}$, fixed L.

Optimised Parameters: α=0.78, L=1.16, δ=0.58, $B(r)$=4.5

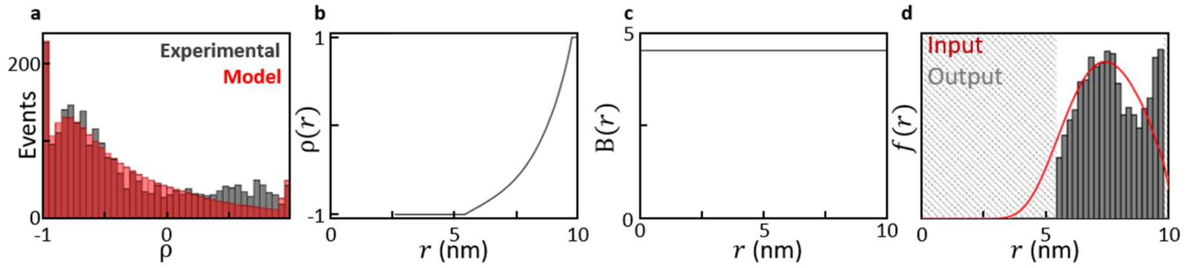

Figure S24 | Optimised Model. a, Histogram of modelled ρ optimised to experimental distribution. **b,** Optimised ρ with radial distance from facet centre r. Undefined where not present. **c,** Model $B(r)$. **d**, Model $B(r)$ (Input) against inverted distribution from optimised model (Output). Inversion undefined where shaded.

x) Optical Model. $B(r)$ = sigmoidal, $W = (1 + L)^{-1}$, fixed L.

Optimised Parameters: α=0.76, L=1.16, δ=0.39, $B(0)$=2.29, $B(R)$=1.07, C=0.20 nm

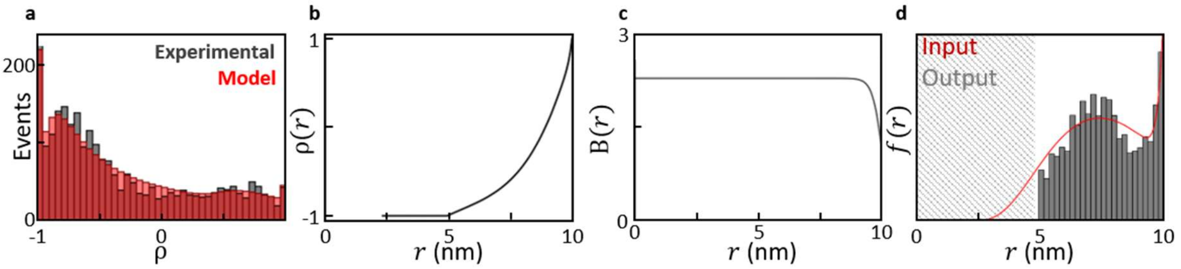

Figure S25 | Optimised Model. a, Histogram of modelled ρ optimised to experimental distribution. **b,** Optimised ρ with radial distance from facet centre r. Undefined where not present. **c,** Model $B(r)$. **d**, Model $B(r)$ (Input) against inverted distribution from optimised model (Output). Inversion undefined where shaded.

xi) Optical Model. $B(r)$ = constant, $W = \left(1 + \alpha^2 L\right)^{-1}$, fixed L.

Optimised Parameters: α=0.81, L=1.16, δ=0.47, $B(r)$=2.26

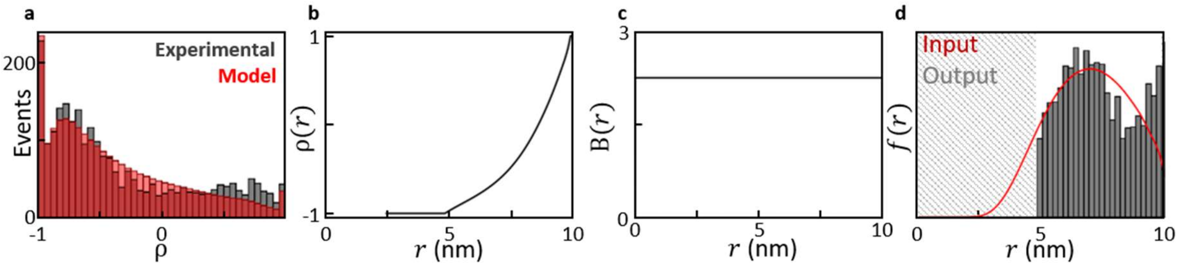

Figure S26 | Optimised Model. a, Histogram of modelled ρ optimised to experimental distribution. **b,** Optimised ρ with radial distance from facet centre r. Undefined where not present. **c,** Model $B(r)$. **d**, Model $B(r)$ (Input) against inverted distribution from optimised model (Output). Inversion undefined where shaded.

xii) Optical Model. $B(r)$ = sigmoidal, $W = \left(1 + \alpha^2 L\right)^{-1}$, fixed L.

Optimised Parameters: α=0.78, L=1.16, δ=0.39, $B(0)$=2.40, $B(R)$=1.18, C=0.29 nm

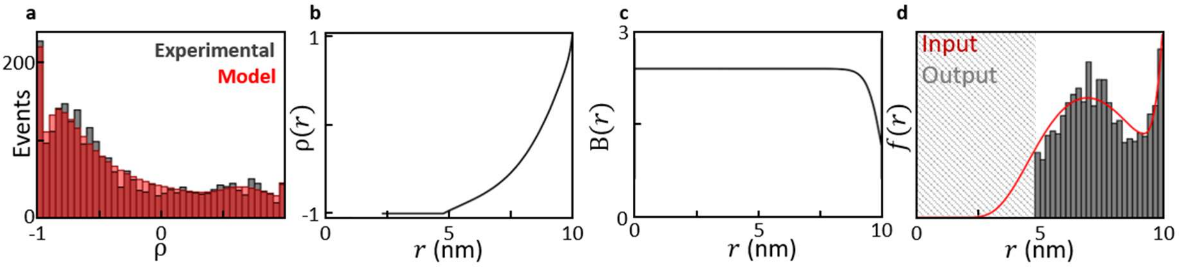

Figure S27 | Optimised Model. a, Histogram of modelled ρ optimised to experimental distribution. **b,** Optimised ρ with radial distance from facet centre r. Undefined where not present. **c,** Model $B(r)$. **d**, Model $B(r)$ (Input) against inverted distribution from optimised model (Output). Inversion undefined where shaded.

Optical Forces

The energy barrier for picocavity formation is known to be ~1 eV (doi: 10.1021/acs.jpcc.5b04383, 10.1021/jp211407p, 10.1126/science.aah5243). This provides an approximate force required to extract an adatom entirely from the bulk gold of ~ 1 eV / 0.3 nm ~ 5 nN. We consider here whether a small atom protrusion (due to a perturbation in the system thermal or otherwise) could lead to a positive feedback optical force and generate a picocavity.

Within the plasmonic cavity, the incident electric field E_0 is enhanced to E. The relative energy of associated with this enhanced field is given by the integral over all space

$$U = \frac{\varepsilon_0}{2}\,|E_0|^2 \int \varepsilon\left(\left|\frac{E}{E_0}\right|^2 - 1\right) dV \tag{19}$$

where ε_0 is the vacuum permittivity and ε is the system relative permittivity.

From FDTD simulations, the system with fully protruded ($\varphi = 2$) picocavity gives a change in optical energy (compared to no protrusion) of 7.5 and -60 neV per µW / µm^2 of incident laser power at 633 nm and 785 nm respectively. These energies are negligible in magnitude compared to the energy barrier for generating an adatom from bulk gold. While the influence of the optical field at 633nm acts against further protrusion, the 785nm field acts with positive feedback but provides a force too small by orders of magnitude. Therefore, such direct optical forces are unlikely to be a significant part of the picocavity generation mechanism.

Fixing L

We use the measured nanocavity SERS ratio for the 633nm and 785nm lasers to estimate their relative near-field intensities. SERS scattered light from within a NPoM cavity couples to the far field through high-angle coupling modes. Therefore, the out-coupling of the NPoM structure is taken as proportional to the square root of the simulated scattering cross section for high angle illumination. This provides an estimate for the average ratio of out-coupling efficiencies for light scattered at the same Raman shift from both the λ_1: 633 nm and λ_2: 785 nm lasers (Figure S28a). This can be used to correct the nanocavity scattering observed for each NPoM for out-coupling efficiency. If the corrected nanocavity emission ratio at λ_2 to λ_1 is defined as R_N, this helps fix L for this large experimental dataset.

$$L = \left(\frac{A_{\lambda_2}}{A_{\lambda_1}}\right)^2 \mathrm{N}_2 \tag{20}$$

$$R_N = \left(\frac{A_{\lambda_2}}{A_{\lambda_1}}\right)^4 \mathrm{N}_4 \tag{21}$$

$$\therefore\ L = \sqrt{\frac{{\mathrm{N}_2}^2}{\mathrm{N}_4} R_N}. \tag{22}$$

This has a mean value of L = 1.16 (Figure S28b).

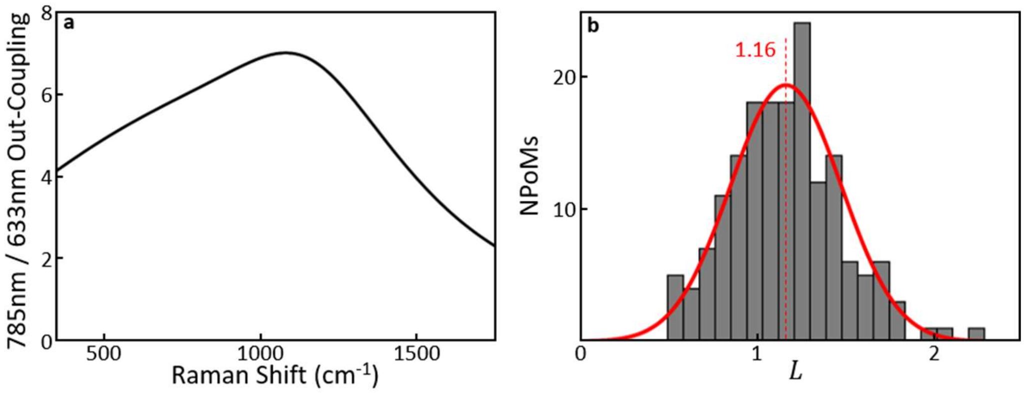

Figure S28 | Fixing L. a, Using the numerically-simulated scattering cross-section vs wavelength with high angle illumination, the out-coupling efficiency ratio for light scattered at the same Raman shift from each excitation wavelength can be calculated. **b,** Corrected for out-coupling, the nanocavity scattering ratio at each scattering wavelength can be converted into the ratio of optical powers coupled into the cavity. The spread of values represents the variation in nanoparticle size and shape compared to the truncated sphere model. This distribution is normally distributed (red line) with a mean of L = 1.16.

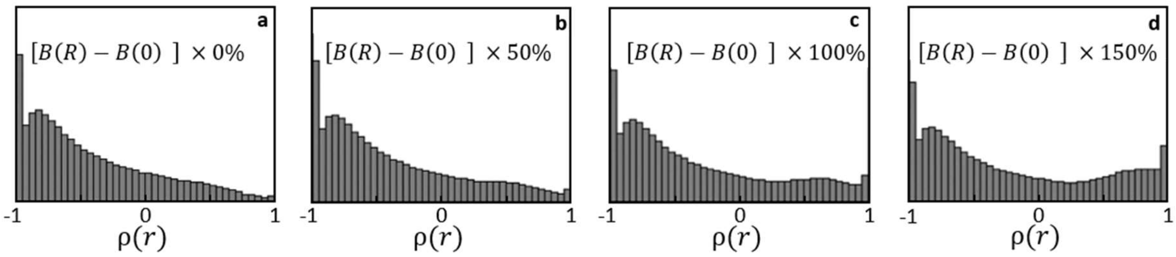

Figure S29 | Varying energy decrease at facet edge. Generated ρ histograms for the (x) "Optical Model. $B(r)$ = sigmoidal, $W = (1 + L)^{-1}$, fixed L" model , when changing the decrease in energy barrier near the facet edge from **a,** 0% to **b,** 50% **c,** 100% **f,** 150% of the optimised value.

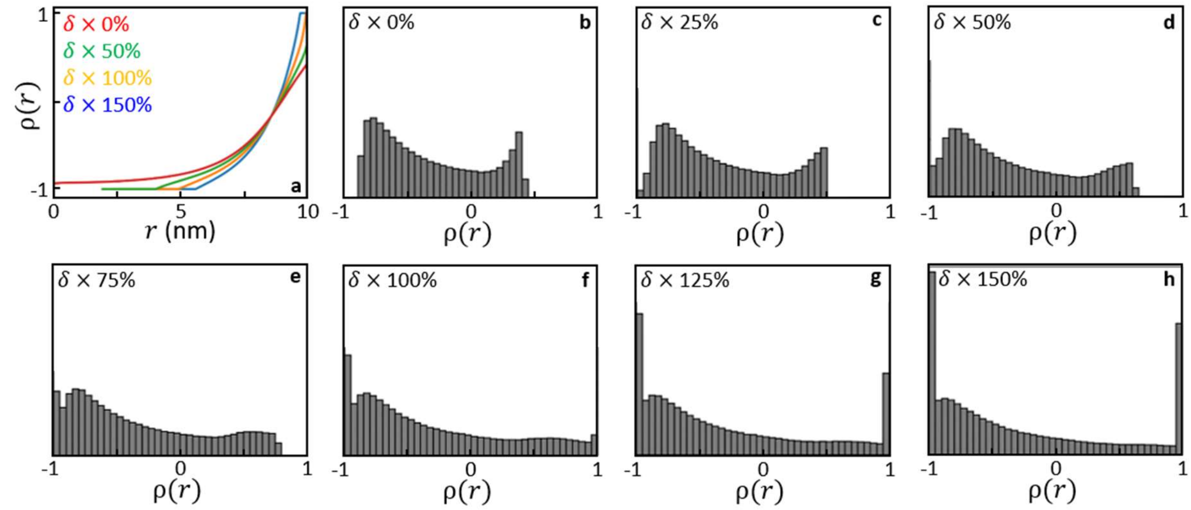

Figure S30 | Effect of scaling δ. a, ρ(r) for the (x) "Optical Model. $B(r)$ = sigmoidal, $W = (1 + L)^{-1}$, fixed L" model , when scaling the noise parameter δ by **b,** 0% **c,** 25% **d,** 50% **e,** 75% **f,** 100% **g,** 125% **h,** 150%.

Extracting Metastable State Energy Difference

In Figure 4 of the manuscript, the system is seen to switch thermally between two states separated by an energy barrier. When the SERS dominates in 633nm or 785nm SERS, the corresponding energy states are labelled as E_1 and E_2 respectively. The energy barrier is given by $E_B > E_1, E_2$ and the difference in energy between the states is defined as $\Delta E \equiv E_1 - E_2$. From the Arrhenius equation, the average lifetimes in these two states $i = \{1,2\}$ are given as $L_i = A \exp\left\{\frac{E_B - E_i}{k_B T}\right\}$ where A is an unknown constant. This constant is removed, along with the barrier energy, using the ratio $R \equiv \frac{L_2}{L_1} = \exp\left\{\frac{\Delta E}{k_B T}\right\}$. Experimentally, samples $\{x_j\}$ and $\{y_j\}$ are extracted for the lifetimes in states 1 and 2 respectively. These are expected to be drawn from exponential Probability Density Functions (PDFs), allowing the PDFs for the underlying values of L_i to be written as

$$P_1(L_1) = \frac{C_1}{L_1} \prod_j e^{\frac{-x_j}{L_1}} \tag{23}$$

$$P_2(L_2) = \frac{C_2}{L_2} \prod_j e^{\frac{-y_j}{L_2}} \tag{24}$$

where C_i are normalisation constants (Figure S31a). The PDF for a ratio R between average state lifetimes is given by

$$P_R(R) \propto \int_0^\infty P_1(\alpha)\, P_2(R\alpha)\, d\alpha \tag{25}$$

(Figure S31b). This can finally be converted to the probability of ΔE using

$$P_{\Delta E}\left(\frac{\Delta E}{k_B T}\right) d\left(\frac{\Delta E}{k_B T}\right) = P_R(R) dR \tag{26}$$

$$P_{\Delta E}\left(\frac{\Delta E}{k_B T}\right) = P_R\left(R = e^{\frac{\Delta E}{k_B T}}\right) e^{\frac{\Delta E}{k_B T}}. \tag{27}$$

Here, this provides a value of (0.97±0.6) $k_B T$ with the width of the PDF arising from the smaller number of lifetime measurements for this dataset (Figure S31c).

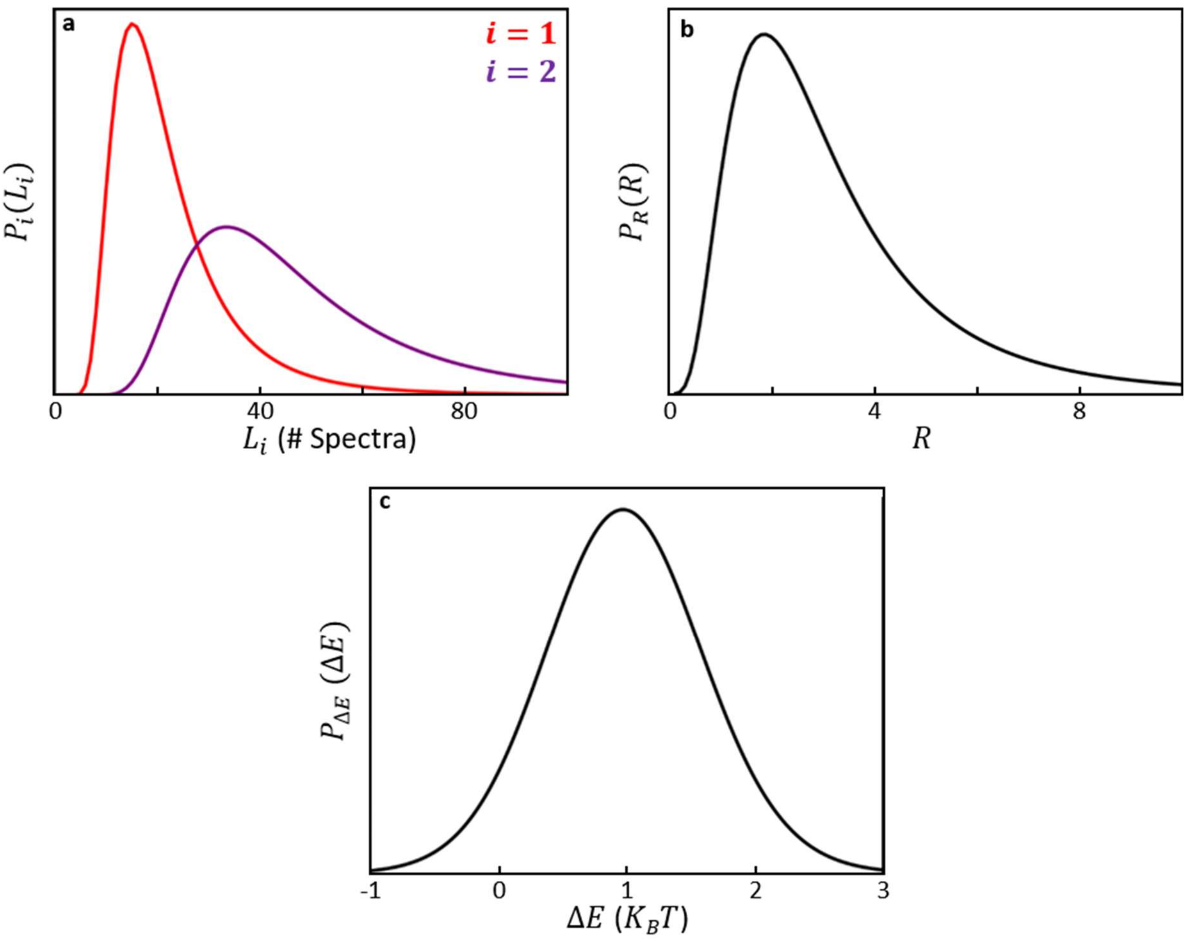

Figure S31 | Calculating ΔE. a, Using sample lifetimes for metastable states 1 and 2, the probability density functions for the underlying mean exponential distribution lifetimes can be calculated. **b,** These are converted into a single PDF for R, representing the mean lifetime of state 2 being R times that of state 1. **c,** This gives the PDF for the energy difference ΔE between the two metastable states.

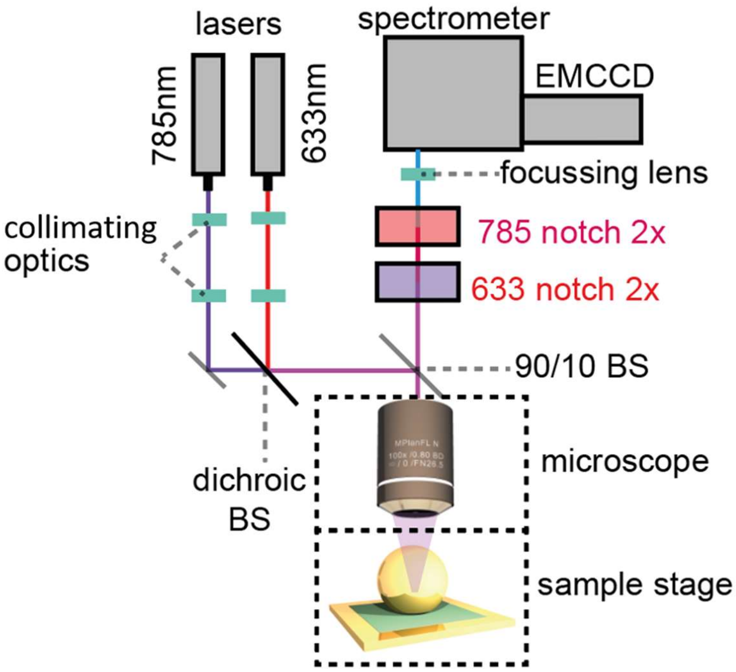

Figure S32 | Experimental set up. Simplified schematic of the custom built experimental system used for SERS collection from individual NPoM constructs with two incident scattering wavelengths.
